# Machine learning tools used for mapping some immunogenic epitopes within the major structural proteins of the bovine coronavirus (BCoV) and for the *in silico* design of the multiepitope-based vaccines

**DOI:** 10.3389/fvets.2024.1468890

**Published:** 2024-10-02

**Authors:** Nithyadevi Duraisamy, Mohd Yasir Khan, Abid Ullah Shah, Reda Nacif Elalaoui, Mohammed Cherkaoui, Maged Gomaa Hemida

**Affiliations:** ^1^College of Science, School of Engineering, Department of Digital Engineering, Computer Science, and Artificial Intelligence, Long Island University, Brooklyn, NY, United States; ^2^Department of Veterinary Biomedical Sciences, College of Veterinary Medicine, Long Island University, Brookville, NY, United States

**Keywords:** BCoV, epitope mapping, B cell and T cell multiepitope, MHC class II molecules, Molecular docking, Toll like receptors, In-silico cloning

## Abstract

**Introduction:**

BCoV is one of the significant causes of enteritis in young calves; it may also be responsible for many respiratory outbreaks in young calves. BCoV participates in the development of bovine respiratory disease complex in association with other bacterial pathogens. Our study aimed (1) to map the immunogenic epitopes (B and T cells) within the major BCoV structural proteins. These epitopes are believed to induce a robust immune response through the interaction with major histocompatibility complex (MHC class II) molecules (2) to design some novel BCoV multiepitope-based vaccines.

**Materials and Methods:**

The goal is achieved through several integrated *in silico* prediction computational tools to map these epitopes within the major BCoV structural proteins. The final vaccine was constructed in conjugation with the Choleratoxin B toxin as an adjuvant. The tertiary structure of each vaccine construct was modeled through the AlphaFold2 tools. The constructed vaccine was linked to some immunostimulants such as Toll-like receptors (TLR2 and TLR4). We also predicted the affinity binding of these vaccines with this targeted protein using molecular docking. The stability and purity of each vaccine construct were assessed using the Ramachandran plot and the Z-score values. We created the *in silico* cloning vaccine constructs using various expression vectors through vector builder and Snap gene.

**Results and discussion:**

The average range of major BCoV structural proteins was detected within the range of 0.4 to 0.5, which confirmed their antigen and allergic properties. The binding energy values were detected between −7.9 and −9.4 eV and also confirmed their best interaction between our vaccine construct and Toll-like receptors. Our *in silico* cloning method expedited the creation of vaccine constructs and established a strong basis for upcoming clinical trials and experimental validations.

**Conclusion:**

Our designed multiepitope vaccine candidates per each BCoV structural protein showed high antigenicity, immunogenicity, non-allergic, non-toxic, and high-water solubility. Further studies are highly encouraged to validate the efficacy of these novel BCoV vaccines in the natural host.

## 1 Introduction

BCoV is one of the significant causes of enteritis in young calves; it may also be responsible for many respiratory outbreaks in young calves (1, 2). BCoV participates in the development of bovine respiratory disease complex in association with other bacterial pathogens (1, 3). BCoV infection in young calves leads to diarrhea, marked reduction in the body weight, dehydration, and death if it is superimposed with other viral pathogens, particularly rotavirus and other bacterial pathogens such as *Mannheimia haemolytica* and some other parasitic diseases, particularly cryptosporidium (4). BCoV belongs to the Betacoronavirus along with other essential coronaviruses affecting humans, such as the severe acute respiratory syndrome-2 (SARS-CoV-2), the Middle East Respiratory Syndrome coronavirus (MERS-CoV), and the human coronavirus-OC43 (HCoV-OC43). This viral genome is a single-strand positive sense RNA approximately 31 kb in length. The viral genome is flanked by two untranslated regions at both ends. The 5′ two-thirds of the genome contain Gene-1, composed of two overlapping open reading frames (ORF1a and ORF1b) with ribosomal frameshifting sites in between. Gene-1 is further processed into 16 non-structural proteins (NSPs; NSp1-NSP16). The 3′ end of the genome encompasses the major structural proteins (S, HE, M, and N). The BCoV genome is organized as follows (5′UTR-Gene-1, S, HE, M, E, N-UTR-3′). Within the five structural proteins, the two proteins play a major role in viral attachment and causing infection, such as S spike and N nucleocapsid protein, whereas HE supports in entering and attaching the host cells, E involves in assembling and releasing of virus particles, and M the abundant structural protein that shapes the new budding viruses. Thus, studying about these proteins by designing the vaccines along with their properties will be effective to prevent the cause of viruses in both cattle and humans.

As a severe disaster caused due to this COVID-19 and their mutations, we as the researchers are in state of producing solution to the social impact to suppress/prevent this deadly disease. Though researchers and clinicians have developed several generations of vaccines, there are more circumstance causes in terms of immersive immunization. Another common challenge in the coronavirus vaccinology is the continuous change of the genetic makeup of coronaviruses over time through mutations, recombination, and new host adaptations. These factors contributed to the vaccination failure of conventional vaccines. Specifically for bovine, only a limited number of vaccines are commercially available in the market. They are mainly based on old technology that uses the live attenuation approach, where immunization is produced through the replication of a group of viruses without causing severe diseases and it is one of the oral medications for young calves. The major disadvantages of the live attenuated-based coronavirus vaccines are the reversion to virulence and the possibility of recombination of some field isolates. Hence, to overcome those obstacles, there is a critical necessary to develop some novel BCoV vaccines that match the currently circulating field strain of this virus and offer a good immune response in the vaccinated cattle population. In recent times, most of the researchers turned their pathway in designing multiepitope-based vaccines approach, to map the immunogenic epitopes across the viral genome. This cutting-edge immunoinformatic method was used to build a vaccination with several binding epitopes from B cell and T cell. To be a potential medication candidate, it should meet the following essential characteristics: (i) should have overlapped B-cell, CTL, and HTL epitopes; (ii) should be water-soluble, immunogenic, antigenic, non-toxic, and non-allergic. Several multiepitope-based vaccines have been designed and tested for many viral diseases affecting humans, particularly SARS-CoV-2 (5–11) and various species of animals and birds (12–17).

To be part of bovine coronavirus (BCoV) vaccine prediction, our main goal of this research was to develop some novel multiepitope-based vaccines for all the structural proteins (HE, S, E, M, and N) through *in silico* approach. Initially, it begins with predicting and analyzing B-cell epitopes, and the prediction of T-cell epitopes and binding affinities toward the MHC class I and II molecules was performed and studied. Second, the characteristic features such as the antigenicity, non-toxicity, and non-allergic nature were analyzed through VaxiJen pro, ToxinPred, AllerTop 2.0, and mapping the epitopes via the immune epitope database (IEDB). The importance of anticipating the linear B cell is to produce antibodies, which are critical for inducing a humoral immunological response. The other candidates such as HTLs that recognize foreign antigens and activate B and cytotoxic T cells cause the immune system to eradicate pathogenic microorganisms. The identification and evaluation of the helper T-cell epitope binding to MHC class II molecules are same as that of CTL epitopes and B-cell epitopes as it also plays a vital role in generating antibodies such as B cell and macrophages in turn eliminating the viral infection.

Third, the construction of vaccine was performed with the support of the adjuvants, PADRE (Pan HLA-DR reactive epitope)—the Universal T-helper epitopes and the linkers, which plays a role. The latter is considered a simple carrier epitope, useful in making synthetic or recombinant vaccines, and is known to enhance the response when coupled with adjuvants, while the former one (adjuvants) such as cholera toxin subunit-B (CTB; PDB ID 1XTC) and 50S ribosomal protein L7/L12 (NCBI ID: P9WHE3) empower vaccines to elicit higher levels of antibody production, thereby facilitating a decrease in the antigen dosage required for optimal immunization outcomes (5, 6). In addition, the linkers can give flexibility and stabilize globular conformation while preserving the vaccine construct’s structural integrity.

Fourth, the binding stability between the vaccine construct and the immune receptors was investigated. The molecular docking analysis was conducted between the vaccine of all the structural proteins (HE, S, E, M, and N) of BCoV and Toll-like receptors (TLR2 and TLR4). The Toll-like receptors have the potential to initiate an immune response with the production of inflammatory cytokines when they detect the conservative pathogens associate molecules on the range of microbes such as bacteria virus (7). With reference to the previous research study, several studies show the impact of Toll-like receptors in enhancing the immune response of SARS-CoV. The molecular docking analysis was performed with the help of Biovia Discovery Studio, and the details of the interaction between the amino acids were analyzed using the PDBSum computational tool. Following with docking, the immune simulation was done with ImmSim and the structural modeling using AlphaFold2 (8, 9). *In silico* cloning was performed through Snap gene and vector builder tool to mimic the vaccine construct for *E. coli* strain.

This multidisciplinary approach not only enhances the efficiency of vaccine development process but also offers a strategic pathway for generation of targeted immunogenic responses against BCoV. The finding from this study presents promising epitope candidate that merits further experimental validation, potentially leading to the development of a next-generation BCoV vaccine with improved efficacy and safety profiles.

## 2 Materials and Methods

**Figure 1:**
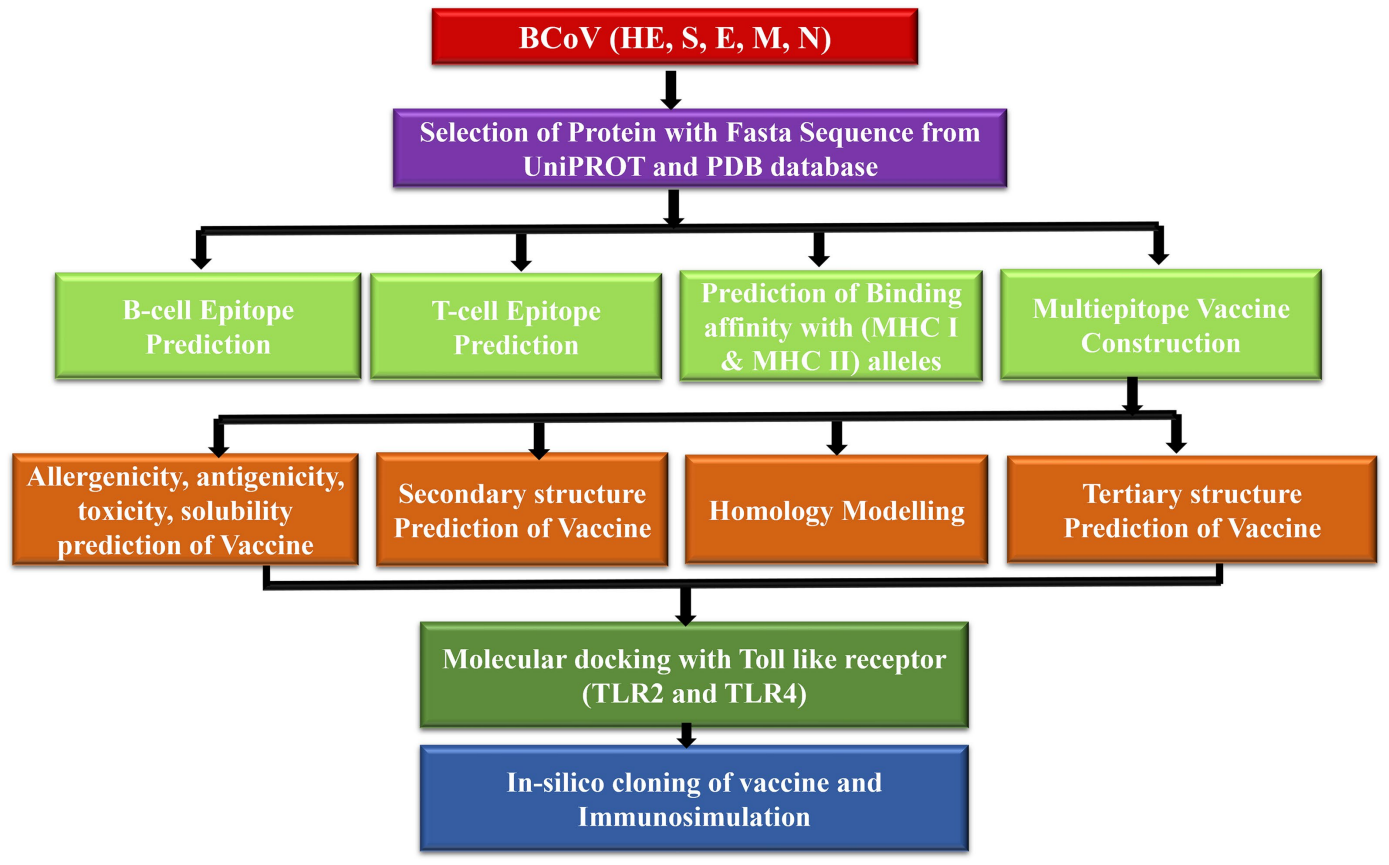
Proposed model workflow for predicting of the linear B-cell and T-cell epitopes and the docking analysis of the binding interactions of these potential vaccine constructs with the TLR complex.

Figure 1 demonstrates the methodology of predicting the epitopes from the desired protein sequence (S spike, N nucleocapsid, M membrane, E envelop, and HE hemagglutinin-esterase protein) followed by the analysis of its characteristic features and checking the interaction with the Toll-like receptors (TLR2 and TLR4) (10) to enhance its immune response with the binding energy value from molecular docking analysis. The immune simulation is used to confirm the type of binding contact and its attributes through their immunogenic properties (11).

### 2.1 Retrieval of the BCoV structural protein sequences

Various BCoV structural protein sequences were retrieved from the NCBI database.^1^ The unique IDs of the BCoV structural proteins are the hemagglutinin-esterase protein HE-3CL5 (PDB id), the spike glycoprotein S-P15777, the nucleocapsid protein N-P10525, the membrane proteins M-P69704, and the envelope protein E-P15779, which were obtained from the Uniport website.^2^ We used the VaxiJen v2.0 and the ANTIGEN pro server to evaluate the protective features of the examined BCoV structural proteins using a threshold value of 0.4 as described elsewhere. Each BCoV was subjected to a rigorous analysis using various bioinformatics tools and several parameters, including the architecture of the major domains, the conservation, and the homology among various BCoV isolates. The comprehensive analysis of individual BCoV structural proteins sheds light on their genetic structures and evolutionary relationship with various BCoV isolates and other coronaviruses. We used the TMHMM online tools to identify the transmembrane helices ™ per each BCoV structural protein. We assigned a value (0–1) as a marker for the good TM helix as previously described (12).

### 2.2 Prediction and mapping of B-cell and T-cell epitopes within the major structural proteins of the BCoV

The default parameters of the IEDB program^3^ were applied as described elsewhere (13) and used to predict the B-cell epitopes within the major structural proteins of the BCoV (HE, S, E, M, and N). It was concluded that all the residues containing five amino acids or more and scores over the cutoff point of 0.5 are associated with an individual epitope. The predicted B-cell epitope sequences above were used to predict the CTL and HTL epitopes by using the Immune Epitopes Database (IEDB). The epitope ranking score is used to predict them; the lower the value, the better the binding affinity.

### 2.3 Evaluation of the major physicochemical properties (antigenicity, allergenicity, toxicity, solubility, and immunogenicity) to design the BCoV multiepitope-based vaccine candidates

In brief, to assess the safety and effectiveness of the vaccine candidates, the ideally designed vaccine must meet the following requirements: It must be antigenic, non-allergenic, non-toxic, and water-soluble. Hence, the physicochemical properties of the designed multiepitope-based BCoV vaccines were assessed using various bioinformatics software as previously described. The toxicity and antigenicity nature of the constructed vaccine were evaluated with the VaxiJen and Antigen servers as previously described for the SARS-CoV-2 vaccine candidates (14). In addition, the AllerTop and the MHC immunogenicity servers were used to evaluate the immunogenicity and the allergenicity of the designed BCoV vaccine candidates as described (15).

### 2.4 Designing of the potential BCoV multiepitope-based vaccines from the major structural proteins and the confirmation of their structural arrangement

**Figure 2:**
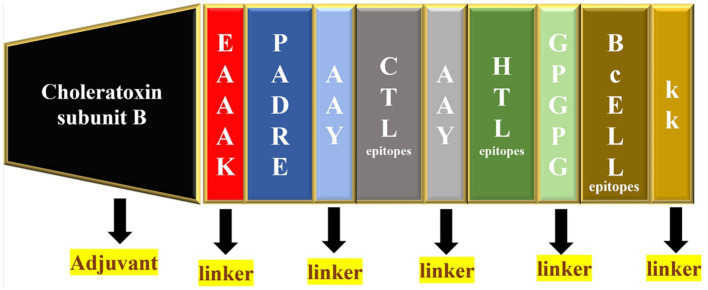
Schematic model of the design of the BCoV (B cells and T cells) multiepitope-based vaccine using the PADRE as linkers and the Cholera toxin subunit B as an adjuvant.

Both the NetMHCpan-4.1 and the NetMHCIIpan-4.0 online tools were used to predict the binding affinity of the BCoV antigen of interest with the cellular MHC class I and MHC class II as previously described (16). The cholera toxin subunit B was selected as an adjuvant for the potential BCoV vaccines to enhance and sustain the immune response. This adjuvant is linked to the N-terminal domains of the designed vaccine candidates using some EAAAK linkers. We also used the AAY to link the universal T-helper epitope (PADRE or TpD) to the designed multiepitope-based immunogens as previously described (11). Moreover, the KK linkers were utilized to join the LBL epitopes with the CTL and HTL epitopes linked together by the AAY linkers, as described in Figure 2.

### 2.5 Analysis of the secondary and the tertiary structures of the designed multiepitope BCoV-based vaccine candidates

To predict and validate the secondary and tertiary structures of a designed BCoV vaccine candidate, we used several sophisticated computational methods and online tools in this study. Initially, we begin with predicting the secondary structure for the final vaccine construct through PSIPRED v4.0 server;^4^ then, the homology models were made using the Biovia Discovery Studio^5^ and AlphaFold2 Colab,^6^ and the assessments of the Z-score, the RMSD, and the Ramachandran plot analysis were used to validate the models as previously described (17).

### 2.6 Molecular docking and binding affinities of the designed epitope-based vaccine candidates with the cellular immunoreceptors

The molecular docking can assess protein-receptor complex binding analysis. These techniques emerged primarily to evaluate the binding affinity of the potential vaccination candidates with host immune receptors. In this scenario, both the Toll receptors such as TLR2 and the TLR4 were used as host cell receptors, while the designed BCoV vaccine constructs served as ligands. These receptors function as regulatory receptors for viral antigens, and their complexes further initiate the cascades of several signaling pathways that mobilize immune cells to counteract viral infections (10). The vaccine constructs were, therefore, docked to the TLR4 (PDB ID:4G8A) and the TLR2 (PDB ID:2Z7X). The Dassault Systems integrated Biovia Discovery studio server was used to evaluate this protein–protein docking validation procedure. Moreover, the PDBSum was utilized to get the graphical representation of the interactions between the vaccine candidates and the host immune receptors as previously described for other viruses such as SARS-COV-2 and Ebola virus vaccine designs (18).

### 2.7 Applications of the *in silico* immune simulation and computational immunology to evaluate the immunogenic properties of the designed multiepitope BCoV-based vaccine candidates

The *in silico* immune simulation analysis was conducted to analyze the immunogenic property and the interactions between the designed BCoV vaccine candidates with the viral proteins. Utilizing the CIMMSIM v10.1 server,^7^ the machine learning platforms, the immune responses of the designed immunogens at several time intervals were analyzed as previously described (19). The immune stimulation capabilities of the designed vaccine candidate in the simulation and in the production of the downstream immune response, such as the cytotoxic T cells, the helper T cells, the B cells, and the other immune cells, also performed as previously described for the SARS-CoV-2 vaccine design (20). We used the default parameters such as random seed:1234, simulated volume: 10, and the simulation steps 1,000.

### 2.8 Application of a combination of codon optimization and *in silico* cloning for the design of the epitope-based vaccine constructs

To simulate the performance of the potential vaccine candidate into the selected expression system, either the prokaryotic or the eukaryotic systems, we applied the *in silico* cloning approach as previously described (21). Thus, to guarantee effective translation and enhanced protein synthesis, the codon corresponding to the vaccine candidate must be optimized in accordance with the target expression system. The Vector Builder codon optimization program^8^ was used to optimize the vaccine construct’s codon usage for the highest possible protein production in the *E. coli* K-12 strain; then, the Snap gene tools^9^ were used to create the cloning of chimeric vaccine construct of all the structural proteins of BCoV referring pET-28a (+) as the compatible plasmid vector.

## 3 Results and discussion

### 3.1 Prediction of the secondary structures of the BCoV major structural proteins

**Figure 3:**
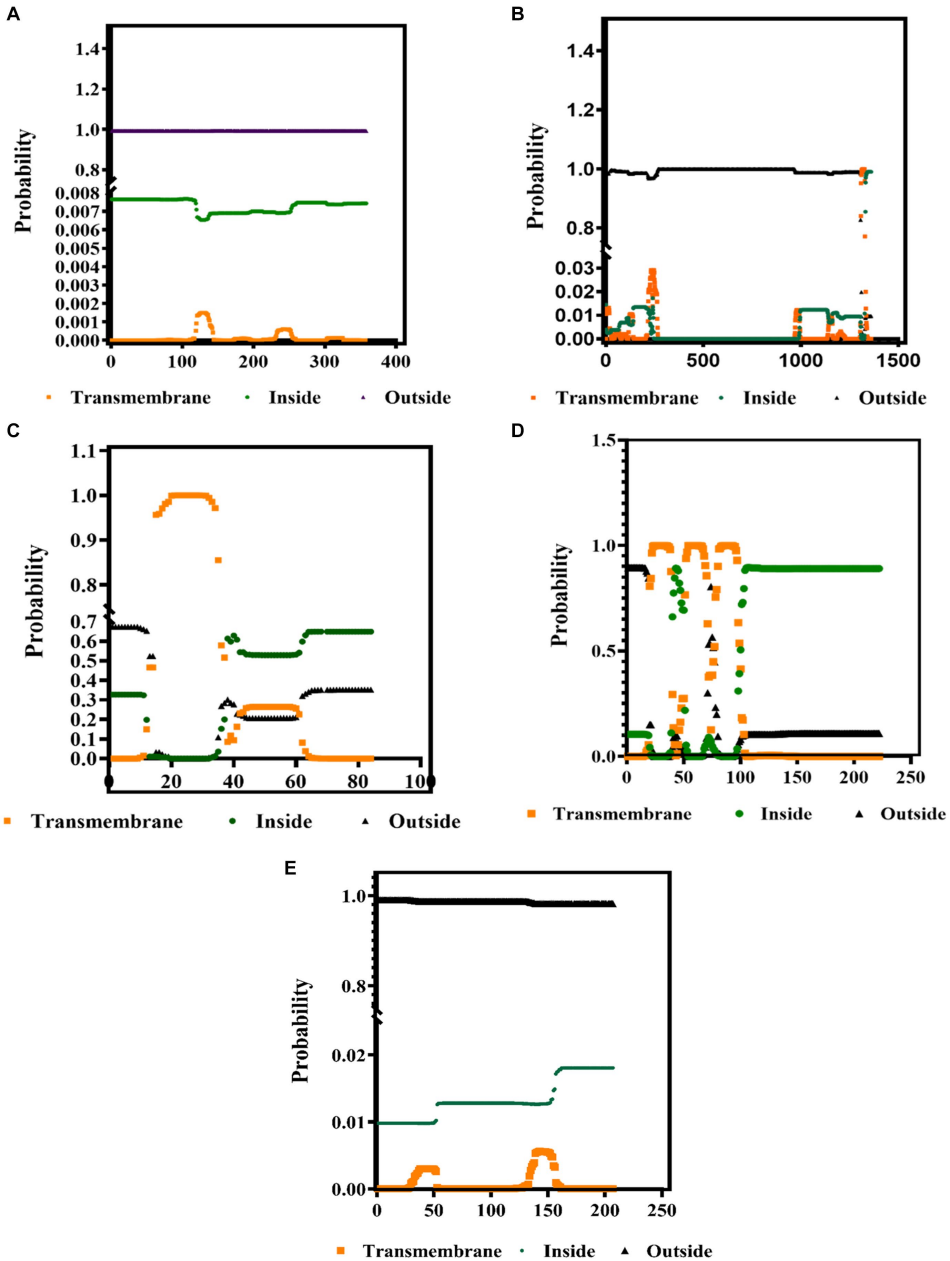
Identification of the transmembrane helices among the BCoV major structural proteins **(A)** HE, **(B)** S, **(C)** E, **(D)** M, and **(E)** N protein using the TMHMM 2.0 computational tools.

The PSIPRED and TMHMM 2.0 were used to predict the secondary structures and the presence of protein in the transmembrane complexes. These tools predict the location of specific amino acids of these proteins, whether the extracellular, the intracellular, or on the surface membrane. Our results showed that the secondary structure of all the structural proteins consists of the alpha helix (pink color), beta-strand (yellow), and the coil turns (gray), shown in Supplementary Table S1. As from the previous reference, the range (0 to 1) denotes the suitable transmembrane helices, which is also confirmed by the results presented in Figures 3A–E. The color codes denote the following: black: the transmembrane, green: intracellular, and orange: outside the membrane (extracellular).

### 3.2 Prediction of B-cell and T-cell (MHC class I and MHC class II) epitopes within the BCoV major structural proteins

#### 3.2.1 The B-cell epitope prediction and assessment

**Figure 4:**
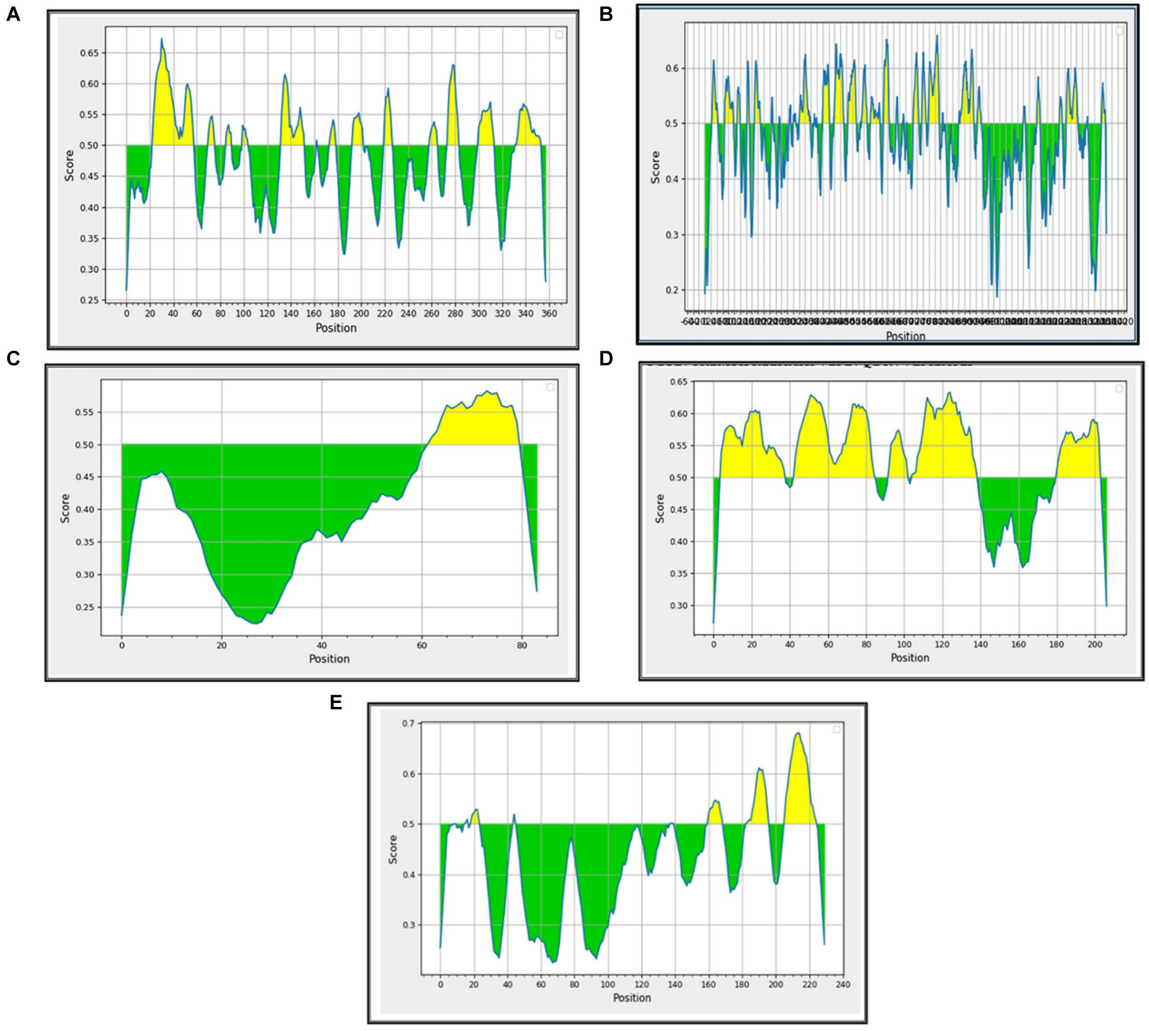
Prediction of B-cell epitopes among the BCoV major structural proteins: **(A)** HE, **(B)** S, **(C)** E, **(D)** M, and **(E)** N.

B-cell epitopes are important components in many vaccines because they are responsible for triggering the humoral immune response in the immunized/infected hosts. They also potentiate the stimulation of the cytotoxic T cells. The linear/continuous B-cell epitopes were predicted for the major BCoV structural proteins with the help of the Immune Epitope Database (IEDB) computational tool using the default parameters. Each B-cell predicted epitope consists of more than five amino acids and was constructed and shown in Supplementary Table S2. The list of these potential B-cell epitopes was used as the input source for predicting the T-cell epitopes, including the classes of MHC (Class I and Class II). The interpreted graphical results (Figures 4A–E) show that the green color peaks represent the presence of epitopes, and the yellow color peaks show the non-epitope sequences across the structural proteins of BCoV.

#### 3.2.2 Prediction of the T-cell epitopes within the BCoV major structural proteins and their binding/interaction with the MHC-I and the MHC-II proteins

##### 3.2.2.1 Cytotoxic T-lymphocyte epitope identification and evaluation

The prediction of cytotoxic T-cell epitopes binding to the MHC class I molecules plays a key role in cellular immunity and in the elimination of viral infections. The epitope prediction analysis started with the application of the identified B-cell epitope sequences as an input into the T-cell binding interaction to MHC class I using the IEBD tool. The epitope predictor version 2023.09 of the NetMHCpan 4.1 EL was used for the Prediction of the alleles (BoLA 1-6, BoLA amani, BoLA T7, BoLA T5, BoLA T2c, BoLA T2b, BoLA JSP.1, BoLA T2a, BoLA HD6, BoLA gb1.7, BoLA D18.4, BoLA, and the AW10) using the default parameters. The predicted epitopes were sorted in a descend peptide predicted score. Confirmation of the epitope prediction was also applied using the IC50 predictions. Once the results were generated, the desired CTL epitopes were filtered initially based on the predicted score of less than or equal to 0.4. The best epitopes were screened, identified, and selected based on their immunogenicity, water solubility, non-allergic, antigenicity, and non-toxic properties.

##### 3.2.2.2 Prediction of the putative helper T-cell epitopes within the major BCoV structural proteins

The helper T-cell epitopes binding to the MHC class II molecules are as important as that of the CTL epitopes and B-cell epitopes. They play a key role in the stimulation of antibody production by B cells. It also activates macrophages to help eliminate viral infections. We started by filtering the epitopes based on the percentile rank of less than or equal to 4 using the IEBD online tools. We used the recommended epitope predictor version 2023.09 of the NetMHCpan 4.1 EL for the alleles of HLA DRB 1-5 (due to the lack of identified cattle alleles, we used similar parameters used for the prediction of the human alleles as previously described). The best epitopes were screened, identified, and selected, as discussed above. Supplementary Table S3 shows the filtered best epitopes of both CTL and HTL binding to MHC class I and II molecules to be used in the design of the downstream vaccine candidate.

### 3.3 Homology modeling and epitope visualization of the potential vaccine candidates based on the identified epitopes from the major BCoV structural proteins

#### 3.3.1 Designing of the potential vaccine constructs based on the identified epitopes in the major structural proteins of the BCoV and their conjugation with the adjuvants

Our criteria for the potential ideal vaccine constructs are (i) the overlapping between the CTL, the HTL, and the B-cell epitopes, (ii) should have the characteristic nature of being antigenic, non-allergic, non-toxic, immunogenic, and water-soluble, and (iii) should have a higher affinity toward the alleles. Based on these criteria, we designed several multiepitope subunit vaccine candidates for the major structural proteins of BCoV. We used the top-ranked [B-cell, T-cell (MHC class I and II)] epitopes and then combined each vaccine candidate with the adjuvants and linkers. Previous research used the universal T-helper epitope Pan HLA-DR reactive epitope (PADRE) to enhance the immune response. The Cholera Toxin B (CTB) adjuvant is the other interesting contender that enhances the immune response by activating both the innate and adaptive immune systems. The main action of CTB in conjugated vaccines is binding to the ganglioside GM1 receptors on the epithelial cell surface. Thus, the CTB adjuvants could be a powerful tool to enhance the efficacy of vaccines by promoting systemic immune responses. They are attached to the N-terminal of the constructed vaccine through the linker EAAK. The next step in the candidate vaccine design was to conjugate the CTL, HTL, and linear B-cell epitopes as a multiepitope vaccine with the adjuvant and PADRE, and the linkers such as AAY for CTL, GPGPG for HTL, and KK for B-cell epitopes are utilized as shown in Table 1.

**Table 1:** Structure and sequences of the designed multiepitope-based vaccine candidates based on the major BCoV structural proteins [Cholera toxin subunit B/EAAAK/PADRE/AAY/CTL/AAY/HTL/GPGPG/B cell/ KK (Adjuvant/linker/PADRE/linker)].

Protein	Final vaccine construct
HE	MSNTCDEKTQSLGVKFLDEYQSKVKRQIFSGYQSDIDTHNRIKDELEAAAKAKFVAAWTLKAAAAAYALCDSGKISSKAAYAQSTALCKAAYFLNKRKDFAAYFRYDNVSSVAAYNKRKDFRWAAYNNARQSDNMAAYRQSDN MTNYAAYRYDNVSSVWAAYSGKISSKAAAYSSKAGNNYAAYTTGFLNKRKAAYVNTNPRNYAAYALCDSGKISSKAGNNGPGPARQSDNMTNYVGVYDGPGPGCDSGKISSKAGNNYTGPGPGDAQQGVFRYDNVSSVGPGP GDDTETITTGFLNKRKGPGPGDFRWNNARQSDNMTNGPGPGDINHGDAQQGVFRYDGPGPGDLNPALCDSGKISSKGPGPGDSGKISSKAGNNYTGGPGPGDTETITTGFLNKRKDGPGPGFLNKRKDFRWNNARQGPGP GFRWNNARQSDNMTNYGPGPGCFLSNTKYYDDTETGPGPGGFLNKRKDFRWNNARGPGPGGKISSKAGNNYTGEGGPGPGISSKAGNNYTGEGNFGPGPGITTGFLNKRKDFRWNGPGPGKDFRWNNARQSDNMTGPGP GKISSKAGNNYTGEGNGPGPGKRKDFRWNNARQSDNGPGPGLNKRKDFRWNNARQSGPGPGLNPALCDSGKISSKAGPGPGMDLNPALCDSGKISSGPGPGNNARQSDNMTNYVGVGPGPGNNYTGEGNFTPYSNDGPGP GNPALCDSGKISSKAGGPGPGPALCDSGKISSKAGNGPGPGPRNYSYMDLNPALCDGPGPGRKDFRWNNARQSDNMGPGPGRNYSYMDLNPALCDSGPGPGRSDCNHVVNTNPRNYGPGPGSSKAGNNYTGEGNFTGPGPG SYMDLNPALCDSGKIGPGPGTGFLNKRKDFRWNNAGPGPGTITTGFLNKRKDFRWGPGPGTTGFLNKRKDFRWNNGPGPGVYDINHGDAQQGVFRGPGPGYDDTETITTGFLNKRGPGPGYDINHGDAQQGVFRYGPGPG YMDLNPALCDSGKISGPGPGYSYMDLNPALCDSGKKKRSDCNHVVNTNPRNYSYMDLNPALCDSGKISSKAGNKKNYTGEGKKNFTPYKKSNDIWKKVYNGSAQSTALCKSGSLVLNKKFYLSGCKKFLSNTKYYDDKKTETI TTGFKKLNKRKDFKKRWNNARQSDNMKKTNYVGVYDINHGDAKKQQGVFRYDNVSSVWPLYSYGRCP
S	MSNTCDEKTQSLGVKFLDEYQSKVKRQIFSGYQSDIDTHNRIKDELEAAAKAKFVAAWTLKAAAAAYDKSVPSPLNWAAYKSQSSRINFAAYLGNKRVELWAAYLNDKSVPSPLNWAAYLSDTKVIKKAAYNDKSVPSPLNWAAY RNMALKGTLLWAAYRRFGFTEQFAAYSQSSRINFAAYSAKSDFMSIAAYSTTNLDNKLQHAAYTNLDNKLQHAAYYRNMALKGTLAAYTSKSTGPYKAAYTTNLDNKLQHGPGPGSDVGFVEAYNNLEAQGPGPGDVGFVEAY NNLEAQAGPGPGFEPFTVNSVNDSLEPGPGPGTFEPFTVNSVNDSLEGPGPGPEPITGNKAPDVMLNGPGPGYYPEPITGNKAPDVMKKVSINDVDTGAPSISTDIVDVTNKKYPTSGSTYRNMALKGTLLLSRLWFKPPFLSDKKTKVIKKGVMYSKKTTNLDNKLQKKHTICHPNLGNKRVELWHWDTGKKGVVTKKKSSAKKKSDFMSKKIAPSTGVYEKKDVYRRIPNLPDCNIEAWLNDKSVPSPLNWERKTFSNCNFKKAKKFTCNKKIKKAAKKKGRKV DLQLGNLGYLQSFNYRIDTTKKVSVSRFNPSTWNRRFGFTEQFVFKPQPVGVFTHHDKKFCPCKLDGSLCVGNGPGIDAGYKNSGIGTKKQKKCLCTPDPITSKSTGPYKCPQKKSGLAIKSDYCGGNPCTCQPQAFLGWSVDS CLQGKKHDVNSGTTCSTDLQKSNTDIKKATYYNSKKDYLTNKKSKKKCNYVFNNTLSRQLQPINYFKKADNSTSSVVQTCDLTKKDYSTKRRSRRAITTGYRFTTFEPFTVNSVNDSLEPVGGKKTIGKKDTTQLQVANSLMNG VTLSTKLKDGVNFNVDDINFSPVLGCLGSDCNKVSSRKKLSDVGFVEAYNNKKLEAQAQIDKKKSQSSRINFCGKKYYYPEPITGNKKKAPDVMLNISTPNLHDFKEELDQWFKNQTSVAPDLSLDYIKKIGTKKCDDYTGHQEL VIKT
E	MSNTCDEKTQSLGVKFLDEYQSKVKRQIFSGYQSDIDTHNRIKDELEAAAKAKFVAAWTLKAAAAAYYNDVKPPVLAAYRGRQFYEFYAAYYNDVKPPVLAAYRQFYEFYNDVAAYNDVKPPVLGPGPGQFYEFYNDVKPPVL DGPGPGRGRQFYEFYNDVKPPKKRGRQFYEFYNDVKPPVLD
M	MSNTCDEKTQSLGVKFLDEYQSKVKRQIFSGYQSDIDTHNRIKDELEAAAKAKFVAAWTLKAAAAAYTQKGSGMDTALAAYTGYSLSDTYKAAYRGFLDKIGDTKAAYYSLSDTYKAAYFLKELGTGYAAYKELGTGYSLAAYDTKV GNYRLAAYKGSGMDTALAAYYSLSDTYKRGPGPGIKFLKEKKLGTGYSLSDKKTYKRGFLDKKIGDTKKKVGNYRLPSTQKGSGMDTALKK
N	MSNTCDEKTQSLGVKFLDEYQSKVKRQIFSGYQSDIDTHNRIKDELEAAAKAKFVAAWTLKAAAAAYLQRDKVCLLAAYGSLELLSFKAAYSLNLQRDKVCLLAAYRSLNLQRDKAAYRSLNLQRDKVCLLAAYLLSFKKERSLAAY LQRDKVCLLHAAYLSFKKERSLAAYEELNPSKLLAAYSLELSFKKERSLAAYGMILGSLELAAYLSFKKERSLAAYHPVEPLVQDRVAAYVEELNPSKLAAYFKKERSLNLAAYGVEELNPSKLLAAYLSFKKERSLGPGPGAHPVEPLVQDRVVEPGPGPGHAHPVEPLVQDRVVEGPGPGLLSFKKERSLNLQRDGPGPGSLNLQRDKVCLLHQEGPGPGIPRLLHAHPVEPLVQGPGPGRSLNLQRDKVCLLHQGPGPGLHAHPVEPLVQDRVVGPGPGLLSFKKERSLNLQ RDGPGPGHDFTILEQDRMPKTSGPGPGSLNLQRDKVCLLHQEGPGPGLLSFKKERSLNLQRDGPGPGCHDFTILEQDRMPKTGPGPGELLSFKKERSLNLQRKKSGPISPTNLEMFKPGVEELNPSKLLLLSYHQEGMKKILGSLEL LSFKKERSLNLQRDKVCLLHQESQLLKLRGTGTDTTKKMATSVNCCHDKKFTILEQDRMPKTSMAPILTESSGSLVTRLMSIPRLKKLHAHPVEPLVQDRVVEPILATEP

#### 3.3.2 Prediction of the 3D structures of the designed multiepitope-based vaccines for the major structural proteins of BCoV

The prediction of the tertiary structure of the BCoV candidate vaccines was made using machine learning tools, particularly the AlphaFold2. Both the Ramachandran plot and Z-score were also used to validate the structural quality of the developed model, and the results are displayed in Supplementary Figure S1A–E. The Z-score of the predicted models was used to select the best model, and the presence of residues in the most advantageous parts of the model validates its highest quality and, consequently, the stability and dependability of the antigen design. When used in tandem, Ramachandran plots and AlphaFold homology modeling greatly improve the accuracy and dependability of vaccine design, guaranteeing the production of stable and successful vaccine candidates.

#### 3.3.3 Visualization of the multiepitope (B cell, CTL, and HTL of T cell) of the major BCoV structural proteins

**Figure 5:**
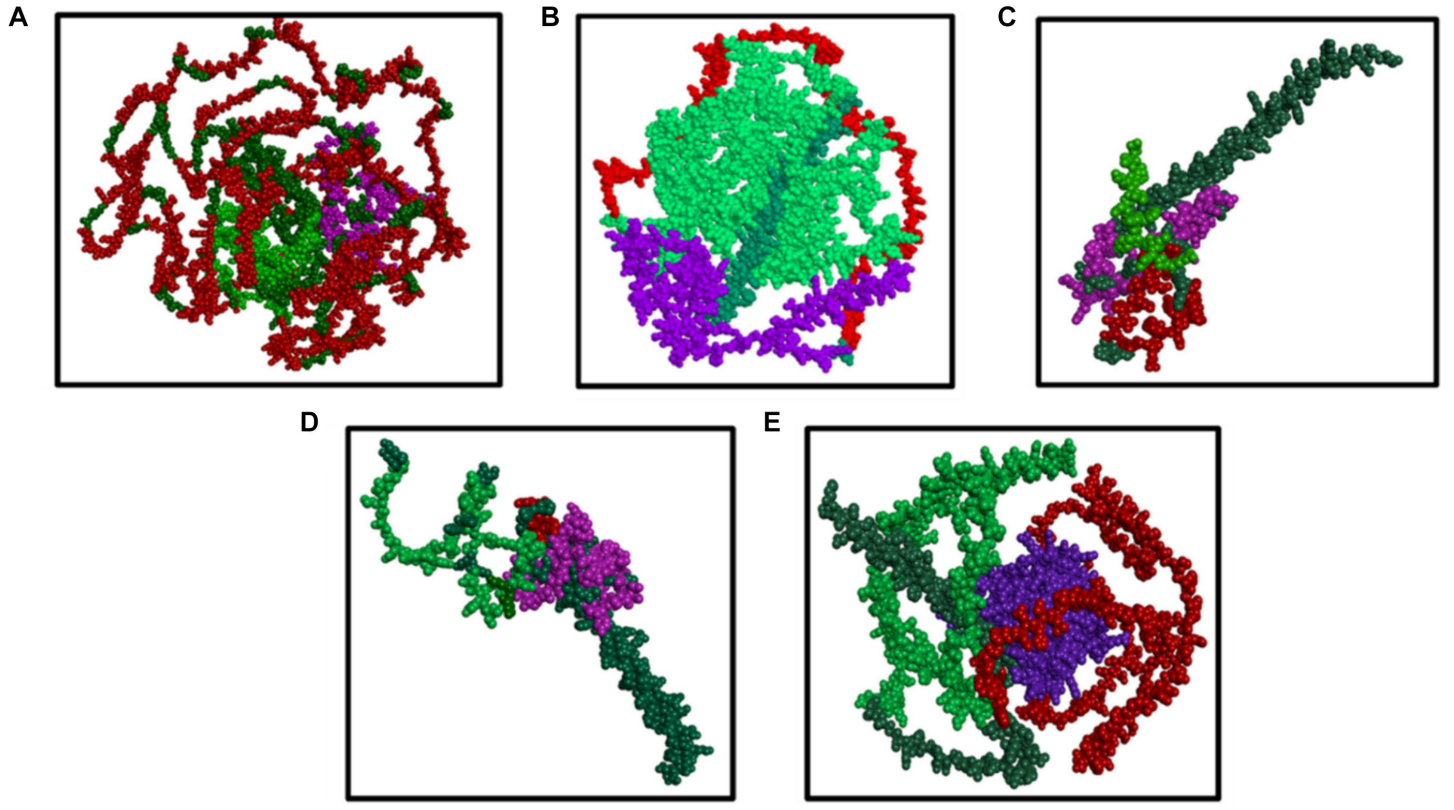
**(A–E)** Results of the visualization of the B cell (green color) and T cell [MHC I (purple color) and MHC II (red color) class] with the predicted epitopes **(A)** S, **(B)** N, **(C)** M, **(D)** E, and **(E)** HE generated by the Dassault system discovery studio tools.

Figures 5A–E visualize the multiepitope vaccine constructs of all the structural proteins (HE, S, E, M, and N) for BCoV through Dassault system Biovia Discovery Studio, which involves the comprehensive analysis and optimization of the structural features of the vaccine design. It involves the following steps: Initially, it starts with importing the desired protein sequence along with adjuvants and linkers into the software using their respective PDB files. The epitope sequences, identified through different computational tools were aligned, and the sequence alignment tool from the discovery studio allows us to map this sequence with their desired corresponding regions.

### 3.4 Results of the molecular docking of the designed BCoV vaccine constructs with the immunoreceptors (TLR2 and TLR4)

**Figure 6:**
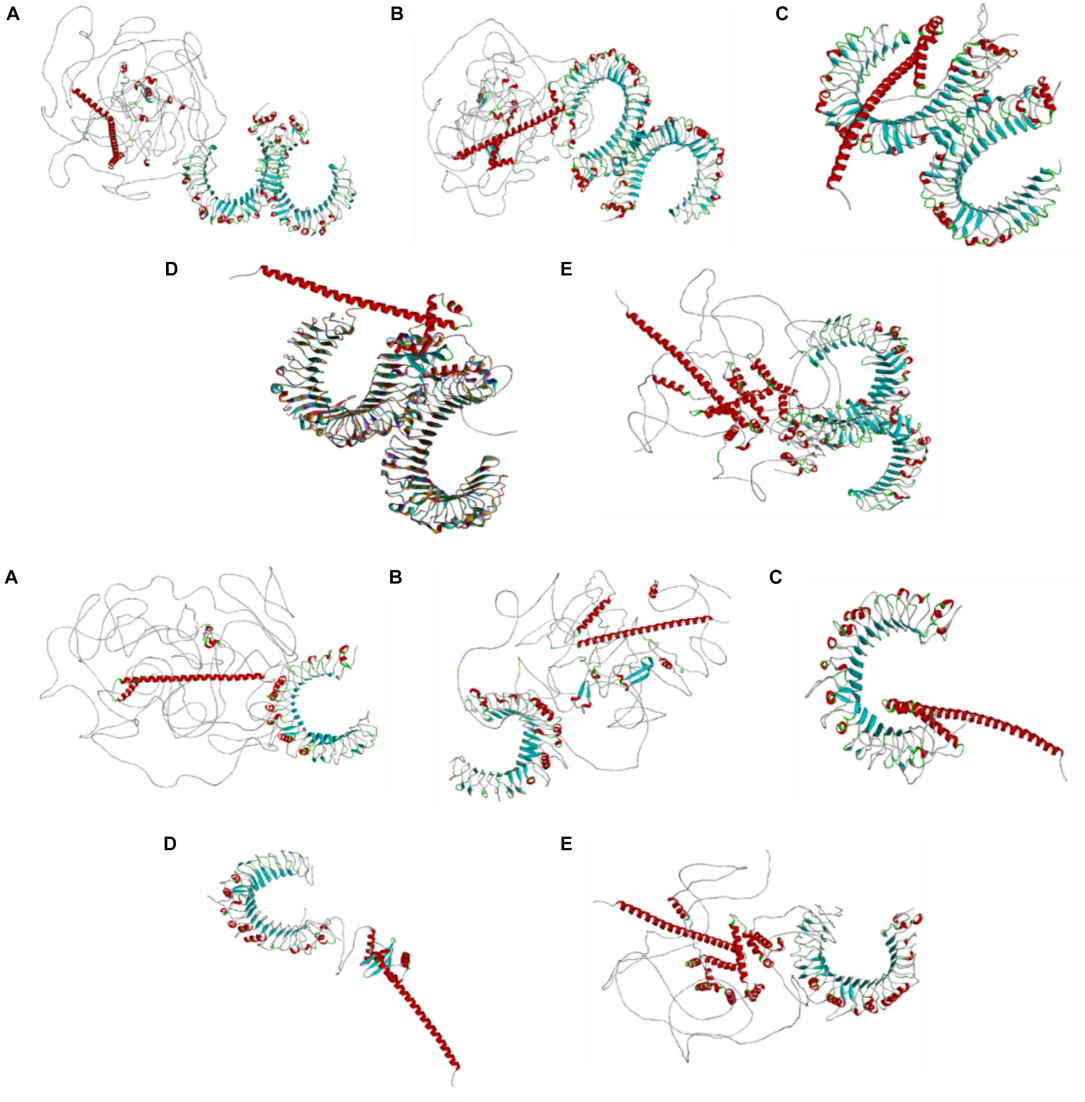
Molecular docking analysis results of the designed vaccine candidates based on the individual structural proteins of the BCoV conjugated with the TLR2 and TLR4 using the Z docker of the Biovia discovery studio, Dassault system.

The results of the docking analysis for the designed vaccine candidates based on the BCoV major structural proteins and the Toll-like receptors (TLR2 and TLR4) are shown in Figure 6. Previous studies showed the impacts of Toll-like receptors in enhancing the immune response of coronaviruses, particularly the SARS-CoV. The molecular docking analysis was performed using the Biovia Discovery Studio, and the details about the interaction between the amino acids were analyzed by the PDBSum computational tool. The results are displayed in Supplementary Figure S2A for the TLR2 and Supplementary Figures S2B,C for the TLR4.

### 3.5 *In silico* immune simulation

Computational tools such as C-ImmSim are widely used to predict the immune response and identify effective vaccine candidates. In these models, we used these tools to predict the immune response of the designed vaccine candidates through the interaction between the BCoV antigens, B cell, T cell, and cytokines. The simulation was performed using the antigen sequence data from the major BCoV structural proteins (HE, S, M, E, and N). The simulation steps were set to 100, while the simulation volume was set to 10. From Supplementary Figure S3(1–5), it is prominent to understand that the vaccine construct of all the structural proteins shows the promising trend of enhanced primary and secondary immune responses against BCoV antigens expressed in our designed vaccine constructs.

### 3.6 The codon optimization and *in silico* cloning of the BCoV multiepitope-based vaccines based on the individual structural proteins

We used the codons matching each of the vaccine candidates; we used the prokaryotic expression system (*E. coli*) to express the target multiepitope-based vaccine candidates described above for the major BCoV structural proteins; THE results are shown in Supplementary Figures S4A–E. We used the vector builder software^10^ to optimize the multiepitope-based vaccine candidates before their insertion into the prokaryotic expression vector. The recombinant plasmids were created by computationally inserting the modified codon-optimized sequences into the pET-28a (+) vector using the Snap Gene program, as shown in Supplementary Figures S5A–E.

## 4 Discussion

BCoV continues to pose a great risk to the two sections of the cattle industry (dairy and beef). Despite the intensive application of some commercial BCoV vaccines, the virus continues to circulate among most cattle populations across the globe, including the USA. The current BCoV vaccine mainly depends on the live attenuation approach, and the vaccination is usually done through the mucosal surfaces in the respiratory tracts. The commercial BCoV vaccines were not tested in pregnant animals (22, 23). There are major factors that hamper the success of most coronaviruses, including BCoV. First, the poor-proof reading capabilities of CoVs. Second, there are possibilities for recombination among various strains/isolates of CoVs. Taking into consideration the dynamic changes of CoVs, including BCoV on the genomic levels, the side effects of the live attenuated vaccines, particularly reversion to virulence, there is a high demand for the design of some novel vaccines that could be upgradable to match the field circulating strains of BCoV at any time. The applications of AI, simulation, and molecular docking paved the way for the development of novel vaccines not only for BCoV but also for other emerging and re-emerging viral diseases. The immunoinformatic approaches using some advanced computational tools could be used to predict and design the multiepitope-based vaccine targeting key structural proteins of BCoV. The usage of this bioinformatics analytical tool to predict the characteristic features of the constructed vaccines, such as antigenicity, allergenicity, and structural validation, alongside molecular docking and *in silico* immune simulation, leads to the development of a vaccine capable of eliciting strong cellular and humoral immune responses, paving the way for the development of some novel BCoV vaccines to protect cattle against the field viral infection (24).

However, it gathers information from immunology, proteomics, and genomes to provide a thorough understanding of pathogen–host interactions and make identifying new targets easier. Furthermore, it facilitates customized treatment by customizing vaccines to each recipient’s DNA profile, improving both efficacy and safety. To our knowledge, the members of BCoV contain the following structural proteins such as spike protein, the genome which is responsible for selection and entry into the target cells, and could be the best candidate for a therapeutic targeted approach, which will pave the way to kill deadly pathogens. Other proteins, such as nucleocapsid protein, are involved in RNA packaging, envelope protein is involved in virus assembly, membrane protein is involved in maintaining viral structure, and the HE protein is involved in viral entry and immune evasion. We adapted several consecutive steps to develop novel BCoV vaccines based on the multiepitope-conjugated approach (25, 26). First, we conducted a multiple sequence alignment of several BCoV isolates using the Mebus strain (accession no: U00735.2) as a reference strain. We predicted the linear B-cell epitopes within the major BCoV structural proteins. Second, we used the predicted B-cell epitopes as input sequences to predict the T-cell epitopes and the binding affinity score for both MHC I and MHC II class molecules. Third, it is essential to analyze the predicted epitopes’ characteristic features as the vaccine construct’s major backbone. The following computational tools have been used to analyze the complexity, structural properties, and characteristic nature of these proteins as well as the epitopes predicted from these protein structures. Tools such as VaxiJen 2.0 and AllerTop were used to study the antigenic properties and allergic nature of the candidates. This could be visualized through the specific range which is from 0.4 to 0.5, indicating the sequence as antigenic, and from this analysis, we confirmed that the selected protein sequences could be the best candidate to be utilized as antigenic mortal (27, 28). We used the Allertop software tools to check and confirm the allergenicity of each vaccine candidate. Similarly, we tested the potential toxicity of each vaccine candidate using the ToxinPred server tools. Once the epitopes meet the assigned requirements to be vaccine candidates, the epitopes, adjuvant, and linkers are built for the major BCoV structural proteins (29, 30).

Fourth, the secondary structures, including alpha helix, beta strands, and coil, were predicted using PSIPRED computational tools. The obtained data demonstrated that the designed vaccine candidates showed a globular, flexible, stable, and stable conformation due to the existence of beta strands, coil turn, and an alpha helix. Fifth, we checked the stability of the designed vaccines using the Ramachandran plot and Z-score. Sixth, the tertiary structure of the designed BCoV vaccines was modeled by the AlphaFold colab2 machine learning algorithms tool. Seventh, the multiepitope-based vaccines were visualized using the Biovia discovery studio, Dassault system. This alignment step is critical as it determines the precise locations of each epitope within the 3D conformation of the designed candidate protein, allowing for precise structural analysis. Each epitope was assigned a color code to differentiate it from the rest and for easy identification and downstream analysis (31). In turn, the amino acids present in the particular region were highlighted to provide a detailed view of the structural context. The representation of the surface was also generated to visualize the accessibility of the epitopes on the protein surface, which is essential for their recognition by the immune system. This will help identify and represent the different categories of the epitopes within the multiepitope vaccine construct, enhancing their potential efficacy. Eighth, the enhanced immune response of our designed BCoV vaccine candidates was tested with Toll-like receptors through binding interaction analytical testing, that is, docking analytical method using the Z docker. In this study, the protein–protein interaction (Zdocker) was analyzed using the built-in protein–protein preparation methods using the Biovia Discovery studio v22.1.021297. The better docking analysis is performed which involves the removal of water molecules, addition of hydrogen, and minimization of energy (CharmM). The docking process results in best 10 different poses; among that, one best pose is chosen with favorable binding energy, which leads to better understanding of interaction between the targeted protein and receptor. To observe the exact binding affinity of the ligand to the protein, the calculation of binding energy is likely an internal step. The binding energy calculations were performed using the Biovia Discovery Studio to define the binding energy of ligands and proteins. The protein data bank provided both the TLR2 and TLR4 receptors with the PDB ID 2Z7X and PDB ID PDB 4G8A. Both the Toll-like receptors crystal structure was determined by the X-ray diffraction with a high resolution of 2.1 Ǻ. Our docking simulation results showed the firm binding affinities between the designed vaccine epitopes and the conjugated TLRs, facilitating effective immune recognition and the initiation of the robust immune response. The molecular docking is a crucial step in the vaccine development. It ensures the specific binding of the designed vaccine epitopes and the target host immune receptors. Below, we discuss the molecular docking results of the designed BCoV multiepitope vaccines based on the major viral structural proteins (32). The Z-score identifies several high-affinity binding poses in the molecular docking results of the designed BCoV-HE vaccines. Our results showed that the best binding pose after refinement has an energy value of-8.3 kcal/mol. Their interaction with these receptors may help the BCoV to evade the host’s immune responses through various mechanisms: (1) altering the signaling pathways, (2) suppressing the immune response, and (3) potentiating the inflammation and tissue damage; the binding of the designed multiepitope vaccine with the TLRs might reduce the receptor availability on host cells, contributing to the viral immune evasion.

The binding analyses of the BCoV-S glycoprotein-designed vaccine candidates with the Toll-like receptors (TLR2 and TLR4) were performed as described above. The PDBsum results showed the interaction between the BCoV/S (chain A) and the TLR2 (chain B). We noticed the presence of 7 hydrogen bonds, three salt bridges, and 343 disulfide bonds. These bonds are called covalent bonds, which ensure stability, structural integrity, and functionality of the generated 3D structural proteins. These disulfide bonds link the different parts of the designed vaccine candidates’ polypeptide chain to maintain the protein’s folded conformation under various environmental conditions (33). The BCoV candidate vaccine-TLR2 interaction residues are Lys505-Sev261, Cys539-Thr247, Ile573-Asp250, Asn550-Lys252, Gln 554-Gly256, and Phe325-Ala274. The surface interaction between the spike protein and the TLR4 is explained, and it is further differentiated into two chain modes as the TLR4 is a dimer molecule. The interaction with the other dimers was listed as Ala 572-Arg86, Ser570-Arg86, Val32-Trp101, His68-Tgr145, Lys57-Tyr69, and Gln91-Gln73. Along with this, a few non-bonded contacts were formed.

The BCoV/E protein is a small, integral membrane protein that plays important roles in molecular pathogenesis, assembly, and release. It is also involved in the modulation of the host’s immune response against BCoV infection. The docking analysis revealed that the E protein of BCoV interacts strongly with both the TLR2 and the TLR4. The best binding pose shows a binding energy of-7.9 kcal/mol and-8.4 kcal/mol. The details about most interaction sites with a disulfide bond, hydrogen bond, salt bridges, and non-contact bond for both TLRs were also studied and identified. The most important key residues involved in this binding/interaction were Asp144, Glu173, and Arg63 for TLR2 and Asp60, Arg87, and Lys165 for TLR4. The docking interaction between the BCoV/M protein and the Toll-like receptors (TLR2 and TLR4) was assessed. The docking simulation was conducted, and the resultant product provides detailed insights into the binding interactions (34, 35). The most favorable binding poses exhibit a binding energy of −8.7 kcal/mol. These results indicate that the BCoV/M protein also has a strong binding interaction with the TLRs.

In confirmation of the docking results, the PDBSum analysis showed the interaction of the active sites between the BCoV/M/TLR2/TLR4. The most prominent active sites for the TLR2 are Asp208, Glu554, and Lys527 which create significant disulfide bonds with the BCoV/M protein. Similarly, for the TLR4, the interactions involved Asp55, Arg98, and Phe101 show the important hydrogen bond interactions. Along with that, disulfide bonds and non-bonded contacts were formed. Thus, based on the results of the binding energy value and active interaction site with hydrogen and disulfide bond, it proves that the BCoV/M protein interaction with the TLRs was efficiently engaged and potentially triggers a robust immune response against the designed BCoV/M vaccine candidate.

We applied the analysis conducted above for various BCoV proteins to the designed BCoV/N candidate vaccine construct with the TLR2 and TLR4. The docking analysis using the ZDOCK showed a strong binding interaction between the designed BCoV/N vaccine constructs and the TLR2 and TLR4. The best pose shows a binding energy of-9.2 kcal/mol. This strong affinity suggests effective engagement of TLRs, which is crucial for robust immune responses. The key residues in this interaction are Asp54, Glu76, and Arg98 on the TLR, forming stable hydrogen bonds and hydrophobic interactions with the designed BCoV vaccine constructs. This highlights the potential efficacy of our designed BCoV vaccine candidates (36, 37). The active interaction site from the PDBSum analysis for both Toll-like receptors and the BCoV/N protein possess; for TLR2, there are 248 disulfide bonds and 13 hydrogen bonds between the chain A (N protein) and chain B (TLR2), whereas, for TLR4, there are 266 disulfide bonds altogether were presented in the chains from A, B, and C and seven hydrogen bonds altogether. The active sites (Pro346-Arg302, Gly349-Glu241, Leu353-Glu215, Gln354-Ile211, Asp344-Gly245) on the TLR2 and the BCoV/N. Regarding the TLR4, the sites were chain A-chain B (Asn352-Met557), Chain B-Chain C (Asn409-Lys435), and the chain A-chain C (Arg343-Lys541, Glu42-Ser391, Asn393-Leu43).

The *in silico* immune simulations using C-ImmSim provided critical insights about the potential immune responses elicited by the designed BCoV vaccine constructs using various structural proteins. The simulations revealed robust activation of T-cell populations, including cytotoxic T cells and helper T cells, crucial for both cellular and humoral immunity. This comprehensive analysis demonstrated that the BCoV vaccine constructs in this study will induce strong humoral and cell-mediated immunity that might play important roles in protecting cattle against BCoV infection.

Based on the Supplementary data, a high level of IgM, IgG1, and IgG2 antibodies and the other immune cells are expected at the time of the administration of the candidate vaccines. It also predicted the progression of the magnitude of the immune response with the progression of the time after administering these candidate vaccines (primary immune response).

There are several classical approaches for the epitope mapping across the viral genomes. The classical method was dominating this field of research for many years such as the application of monoclonal antibodies against some viral immunogens within the BCoV genomes (38). However, this method is lengthy, very laborious, and expensive and requires the application of animals. This contrasts with the application of AI in the prediction and simulation of protein/protein interactions which is very efficient and fast and has high level of accuracy.

*In silico* cloning techniques and codon optimization were used to improve the expression and effectiveness of the candidate vaccines in the prokaryotic expression system. Our approach ensured that the vaccination candidates had reliable protein synthesis and effective translation using the optimized codons and utilizing computational tools such as vector builders and Snap gene (39). The optimized sequence of the designed BCoV vaccine candidates was inserted into an expression vector for the target protein expression. The improved sequence was integrated, and the recombinant BCoV vaccines were cloned using the compatible plasmid vector pET-28a (+). This study aimed to create a viable cloning plan for the various BCoV multiepitope-based vaccine candidates. This method expedited the creation of vaccines and established a strong basis for upcoming clinical trials and experimental validations (40). Based on the presented data above, we believe our designed BCoV multiepitope-based vaccine candidates will be strong and will be used for the next generation of BCoV vaccines.

The application of AI in the mapping the immunogenic epitopes could provide several advantages over the classical conventional prediction methods. (1) AI could help in the advancement of the currently available diagnostic assays such as ELISA by prediction the species-specific epitopes, to design some diagnostic assays that fulfill the DIVA concept (differentiation between infected and vaccinated animals) and (2) to design some broad species spectrum diagnostic assays that enable the detection of BCoV in not only the domestic cattle, but also other feral buffalo and Bison. This will overcome the limitations of the currently available diagnostic assays in the differentiation between the CoV circulating in the domestic/wild bovine population (41, 42).

Our proposed multiepitope BCoV vaccine candidates include some linkers which play important roles not only in the stability of these vaccine candidates but also in the enhancing the immune response against BCoV. Similar approach was adapted in the design of some novel multiepitope vaccines against Brucella in cattle. The linkers were used to connect various epitopes in the multiple design against some major Brucella proteins (BvrR, OMP25, and OMP31) (43). These linkers have several advantages not only on the vaccine development but also on enhancing the immune responses against these multiepitope-based vaccines. The linkers prevent the formation of the non-desired epitopes and enhance the helper T-cell immune response (43).

This study briefly explains the importance of *in silico* methodology for identifying and predicting the validated epitopes to be used as vaccine candidate for bovine coronavirus. Though it provides us with better visibility, there is a chance of fewer limitations that need to consider as well. As we are making use of the existing computational tool platforms for our evaluation and validation, it is indirectly depending on the quality and completeness of our input data and the performance efficiency of the tools we used for our purpose. In addition, though the better validation results in better vaccine candidate, the need of experimental interpretation through *in vitro* and *in vivo* is important and needed before utilizing the predicted epitopes as a valuable vaccine candidate because the vaccine efficacy might be affected on several viral mutations. Even though we see limitations, there is strong proof of evidence for future research with our findings including prediction algorithms, the wide-ranging datasets, and analyzing and collecting the information about the specific properties, binding interaction with immune proteins, and predicting various vectors interaction with *in silico* vaccine cloning, which saves more time and animal resources. With these solutions, the analysis of this study could be potentially contributed to the development of multiepitope-based vaccine candidate for various viral pathogens.

## 5 Conclusion

The construction of multiepitope-based vaccines can be considered as the best ingredients and solution for the worldwide health issues caused through these viral pathogens. Applying conventional technology will provide us the better solution but the resource utilization will be huge which need to be taken into consideration; thus, the implementation of various *in silico* computational tool would provide us the pathway to minimize the time and resources and also would address the issues in smart manner. With this information, the best vaccine candidate could be produced through *in vitro* and *in vivo* studies and can be proceed further to be available as commercial product. Here, in our study, we mapped the main epitopes within the major structural proteins of BCoV. We used these epitopes to design several BCoV multiepitope-based vaccines that show high antigenicity and immunogenicity and are non-toxic and non-allergic. These vaccine candidates are linked with the Cholera toxin B adjuvant and evaluated their binding interaction with strong immunostimulants (TLR2 and TLR4) to ensure a sustained immune response for a long time in the vaccinated cattle against BCoV. The combination of AI tools, including epitope prediction, immune stimulation, and docking, provides a novel approach to vaccine development and reduces the time required for vaccine development and approval.

## Data Availability

The raw data supporting the conclusions of this article will be made available by the authors upon request.

## Footnotes

^1^https://www.ncbi.nlm.nih.gov/

^2^https://www.uniprot.org/uniprotkb/P15777/entry

^3^http://tools.iedb.org/main/

^4^http://bioinf.cs.ucl.ac.uk/psipred/

^5^https://www.3ds.com/products/biovia/discovery-studio

^6^https://colab.research.google.com/github/sokrypton/ColabFold/blob/main/AlphaFold2.ipynb

^7^https://kraken.iac.rm.cnr.it/C-IMMSIM/index.php?page=1

^8^https://en.vectorbuilder.com/tool/codon-optimization.html

^9^https://www.snapgene.com/

^10^https://en.vectorbuilder.com/
