# Supplementary material for: Machine learning tools used for mapping some immunogenic epitopes within the major structural proteins of the bovine coronavirus (BCoV) and for the in silico design of the multiepitope-based vaccines
Source: Front Vet Sci. 2024 Oct 2;11:1468890. doi: 10.3389/fvets.2024.1468890 (PMC11479863; doi:10.3389/fvets.2024.1468890)

| **Table S1: Secondary structure prediction of (a) Hemagglutinin-Esterase protein, (b) spike protein, (c) envelop protein, (d) membrane protein, and (e) Nucleocapsid protein using Psipred server tool** | |
| --- | --- |
| **Protein** | **Secondary Structure** |
| **HE** | 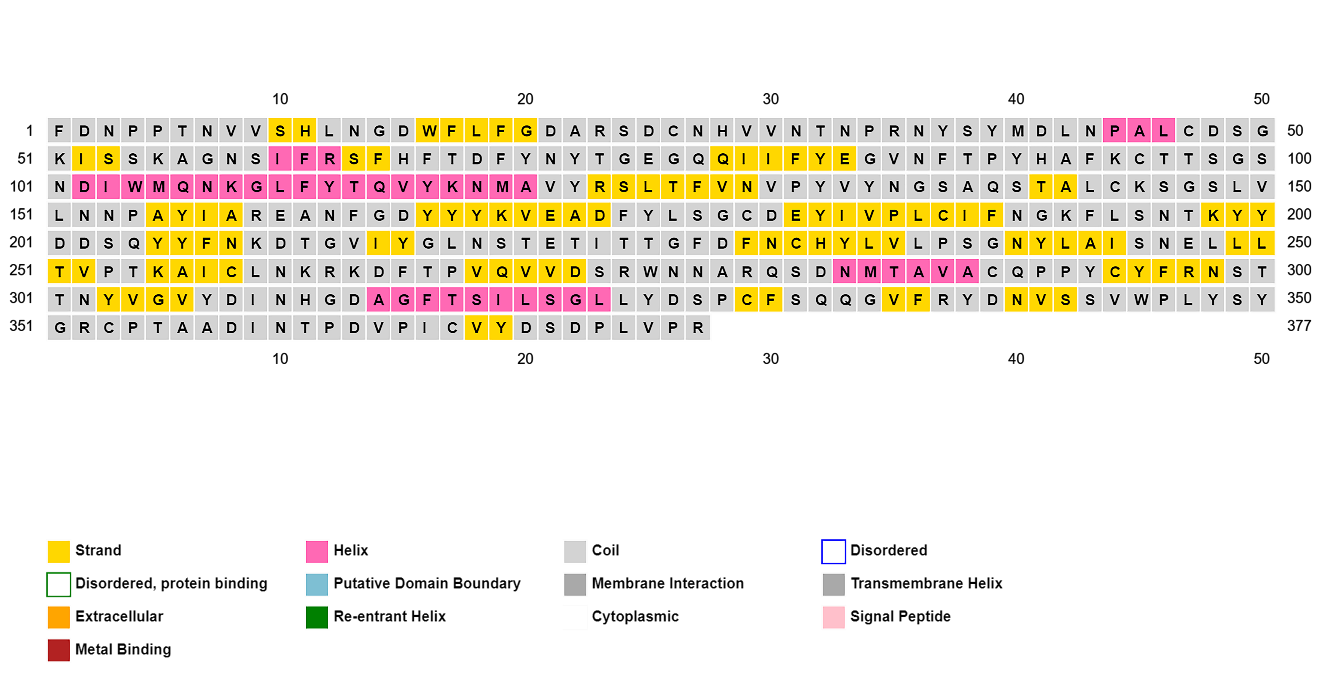 |
| **S** | 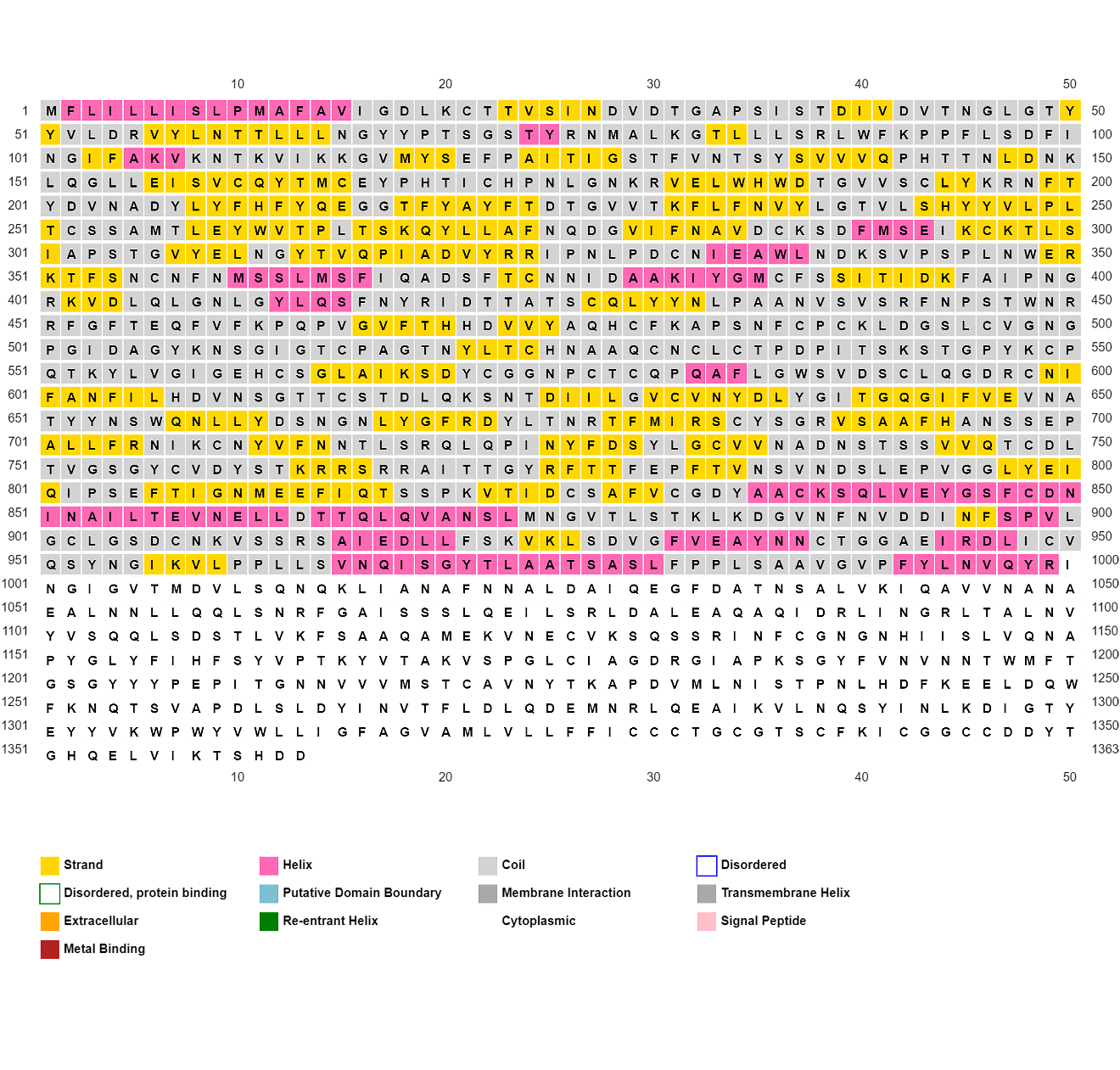 |
| **E** | 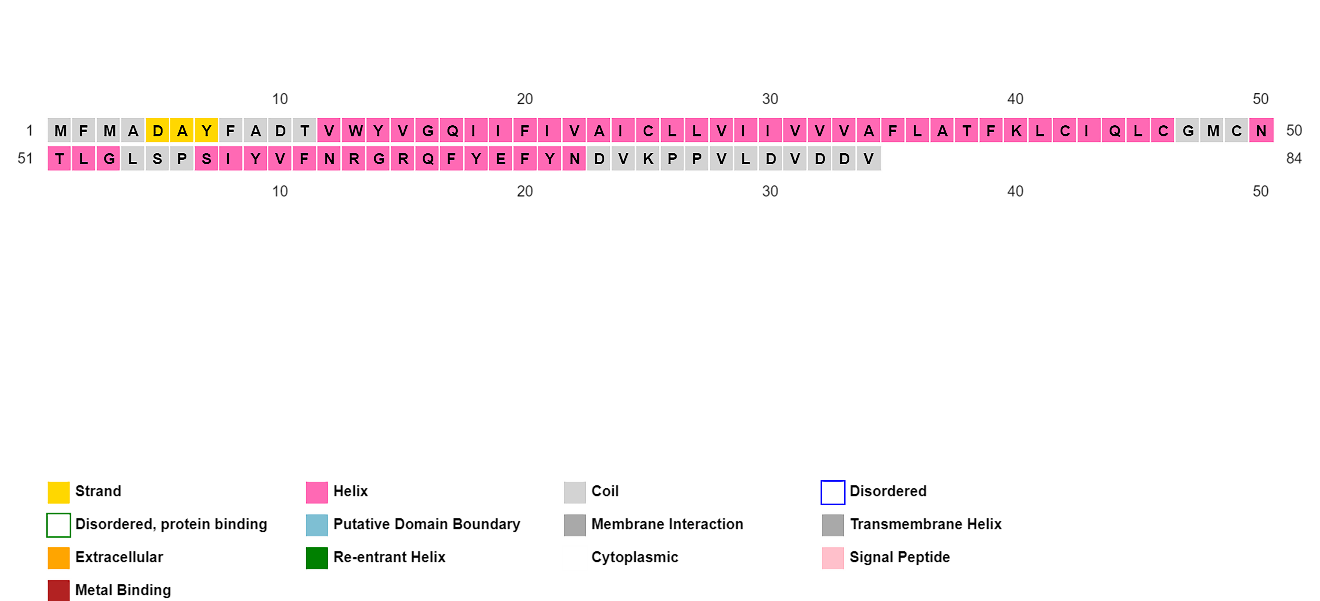 |
| **M** | 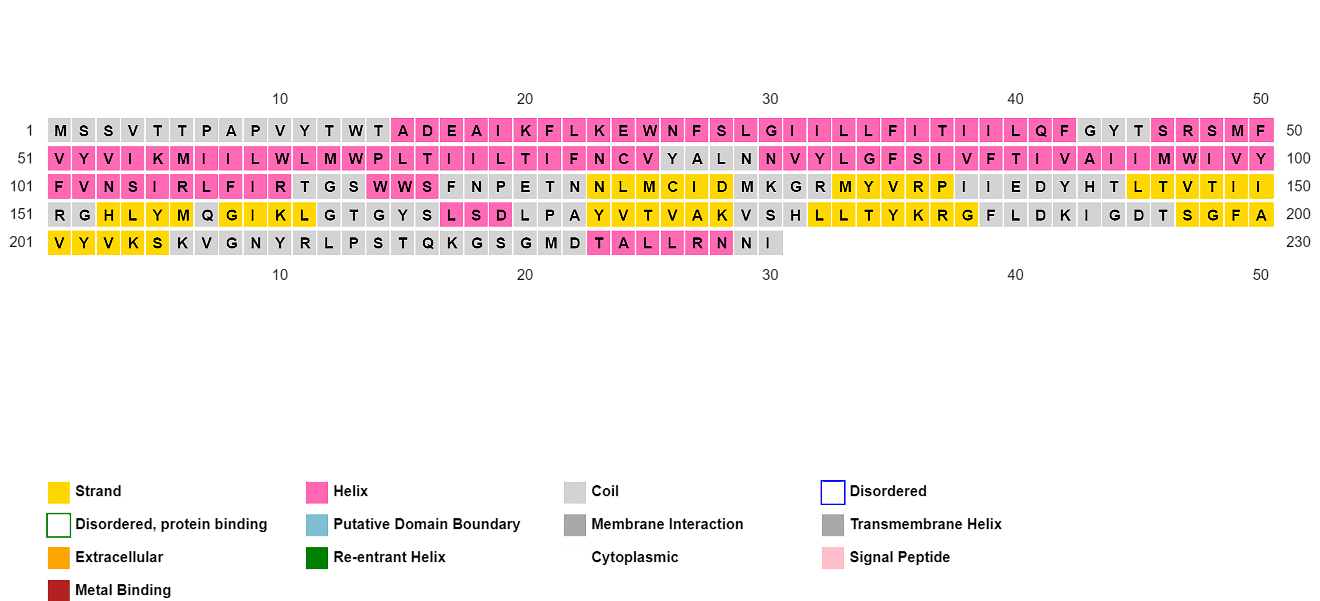 |
| **N** | 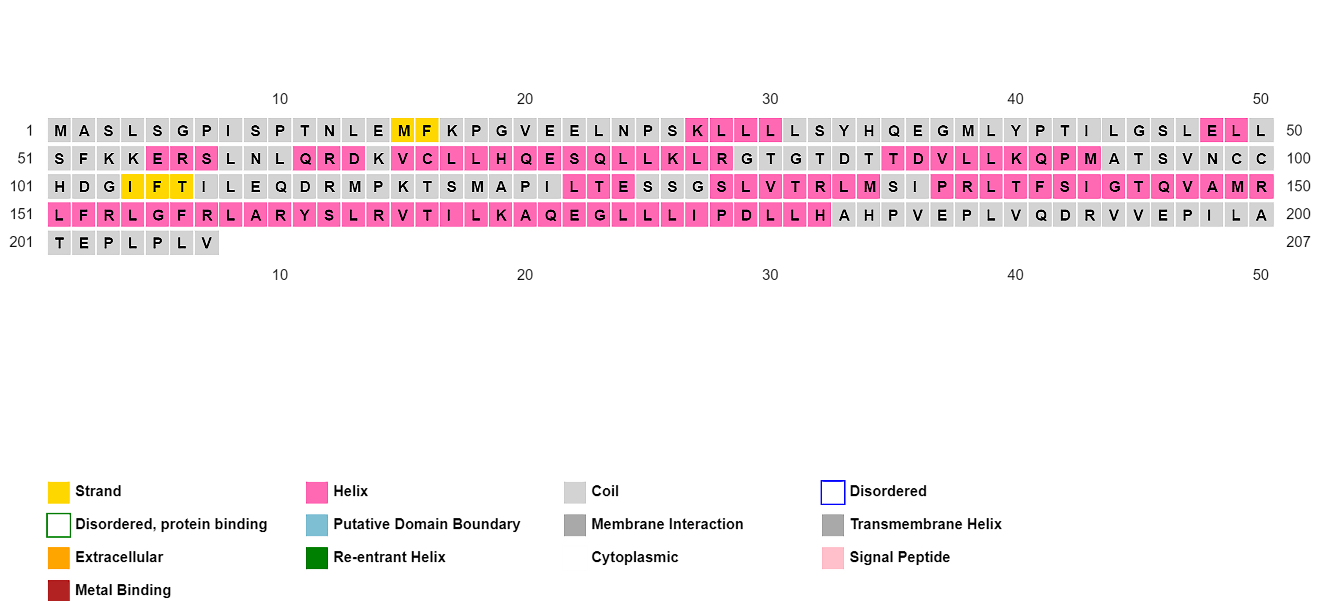 |


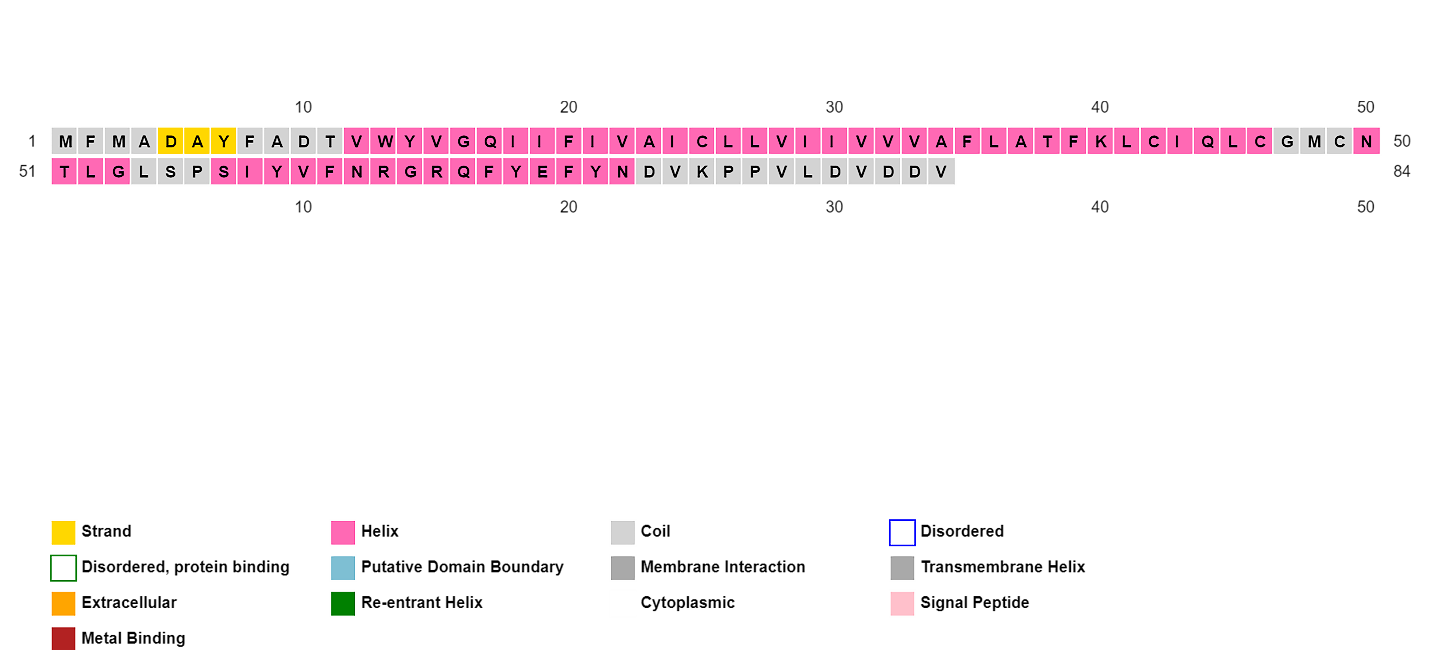

Supplement: Supplementary file 1 [file Data_Sheet_1.ZIP › Supplementary Tables/Table S1.docx]
