# Supplementary material for: Machine learning tools used for mapping some immunogenic epitopes within the major structural proteins of the bovine coronavirus (BCoV) and for the in silico design of the multiepitope-based vaccines
Source: Front Vet Sci. 2024 Oct 2;11:1468890. doi: 10.3389/fvets.2024.1468890 (PMC11479863; doi:10.3389/fvets.2024.1468890)
Supplement: Supplementary file 1 [file Data_Sheet_1.ZIP › Supplementary Tables/Table S2.docx]

| **Table S2. Summary of the information of the shortlisted epitopes used for the design of the candidate vaccine constructs of the major BCoV structural proteins (HE, S, E, M, N) binding with MHC class I and class II molecules.** | | | | |
| --- | --- | --- | --- | --- |
| **HE protein** | | | | |
| **Peptide (MHC I)** | **Antigen/non-antigen** | **Toxin/non-toxin** | **Allergen/Non allergen** | **Solubility** |
| **ALCDSGKISSK** | **0.7049 ( Probable ANTIGEN )** | **Non-toxic** | **PROBABLE non-ALLERGEN** | **Water soluble** |
| **AQSTALCK** | **0.8022 ( Probable ANTIGEN)** | **Non-toxic** | **PROBABLE Non-ALLERGEN** | **Water soluble** |
| **FLNKRKDF** | **1.8948 ( Probable ANTIGEN )** | **Non-toxic** | **PROBABLE Non-ALLERGEN** | **Water soluble** |
| **FRYDNVSSV** | **1.2836 ( Probable ANTIGEN )** | **Non-toxic** | **PROBABLE Non-ALLERGEN** | **Water soluble** |
| **LNKRKDFRW** | **1.8289 ( Probable ANTIGEN )** | **Non-toxic** | **PROBABLE Non-ALLERGEN** | **Water soluble** |
| **NKRKDFRW** | **2.0132 ( Probable ANTIGEN ).** | **Non-toxic** | **PROBABLE Non-ALLERGEN** | **Water soluble** |
| **NNARQSDNM** | **0.8134 ( Probable ANTIGEN ).** | **Non-toxic** | **PROBABLE Non-ALLERGEN** | **Water soluble** |
| **RQSDNMTNY** | **0.7138 ( Probable ANTIGEN ).** | **Non-toxic** | **PROBABLE Non-ALLERGEN** | **Water soluble** |
| **RYDNVSSVW** | **1.0394 ( Probable ANTIGEN ).** | **Non-toxic** | **PROBABLE Non-ALLERGEN** | **Water soluble** |
| **SGKISSKA** | **1.6666 ( Probable ANTIGEN ).** | **Non-toxic** | **PROBABLE Non-ALLERGEN** | **Water soluble** |
| **SSKAGNNY** | **0.5626 ( Probable ANTIGEN ).** | **Non-toxic** | **PROBABLE Non-ALLERGEN** | **Water soluble** |
| **TTGFLNKRK** | **1.7436 ( Probable ANTIGEN ).** | **Non-toxic** | **PROBABLE Non-ALLERGEN** | **Water soluble** |
| **VNTNPRNY** | **1.1707 ( Probable ANTIGEN ).** | **Non-toxic** | **PROBABLE Non-ALLERGEN** | **Water soluble** |
|  |  |  |  |  |
| **Peptide (MHC II)** | **Allergen/Non allergen** | **Toxic/Non-toxic** | **Allergen/Non allergen** | **Solubility** |
| **AGNNYTGEGNFTPYS** | **0.8358 ( Probable ANTIGEN ).** | **Non-toxic** | **PROBABLE Non-ALLERGEN** | **Water soluble** |
| **ALCDSGKISSKAGNN** | **0.6993 ( Probable ANTIGEN ).** | **Non-toxic** | **PROBABLE Non-ALLERGEN** | **Water soluble** |
| **ARQSDNMTNYVGVYD** | **0.4982 ( Probable ANTIGEN ).** | **Non-toxic** | **PROBABLE Non-ALLERGEN** | **Water soluble** |
| **CDSGKISSKAGNNYT** | **0.5579 ( Probable ANTIGEN ).** | **Non-toxic** | **PROBABLE Non-ALLERGEN** | **Water soluble** |
| **DAQQGVFRYDNVSSV** | **0.6134 ( Probable ANTIGEN ).** | **Non-toxic** | **PROBABLE Non-ALLERGEN** | **Water soluble** |
| **DDTETITTGFLNKRK** | **0.8439 ( Probable ANTIGEN ).** | **Non-toxic** | **PROBABLE Non-ALLERGEN** | **Water soluble** |
| **DFRWNNARQSDNMTN** | **0.9422 ( Probable ANTIGEN ).** | **Non-toxic** | **PROBABLE Non-ALLERGEN** | **Water soluble** |
| **DINHGDAQQGVFRYD** | **0.5482 ( Probable ANTIGEN ).** | **Non-toxic** | **PROBABLE Non-ALLERGEN** | **Water soluble** |
| **DLNPALCDSGKISSK** | **0.9845 ( Probable ANTIGEN ).** | **Non-toxic** | **PROBABLE Non-ALLERGEN** | **Water soluble** |
| **DSGKISSKAGNNYTG** | **0.6434 ( Probable ANTIGEN ).** | **Non-toxic** | **PROBABLE Non-ALLERGEN** | **Water soluble** |
| **DTETITTGFLNKRKD** | **0.9567 ( Probable ANTIGEN ).** | **Non-toxic** | **PROBABLE Non-ALLERGEN** | **Water soluble** |
| **ETITTGFLNKRKDFR** | **1.0719 ( Probable ANTIGEN ).** | **Non-toxic** | **PROBABLE Non-ALLERGEN** | **Water soluble** |
| **FLNKRKDFRWNNARQ** | **0.9653 ( Probable ANTIGEN ).** | **Non-toxic** | **PROBABLE Non-ALLERGEN** | **Water soluble** |
| **FRWNNARQSDNMTNY** | **0.4654 ( Probable ANTIGEN ).** | **Non-toxic** | **PROBABLE Non-ALLERGEN** | **Water soluble** |
| **GCFLSNTKYYDDTET** | **0.5617 ( Probable ANTIGEN ).** | **Non-toxic** | **PROBABLE Non-ALLERGEN** | **Water soluble** |
| **GFLNKRKDFRWNNAR** | **1.2905 ( Probable ANTIGEN ).** | **Non-toxic** | **PROBABLE Non-ALLERGEN** | **Water soluble** |
| **GKISSKAGNNYTGEG** | **0.8030 ( Probable ANTIGEN ).** | **Non-toxic** | **PROBABLE Non-ALLERGEN** | **Water soluble** |
| **ISSKAGNNYTGEGNF** | **0.6584 ( Probable ANTIGEN ).** | **Non-toxic** | **PROBABLE Non-ALLERGEN** | **Water soluble** |
| **ITTGFLNKRKDFRWN** | **1.7211 ( Probable ANTIGEN ).** | **Non-toxic** | **PROBABLE Non-ALLERGEN** | **Water soluble** |
| **KDFRWNNARQSDNMT** | **0.8940 ( Probable ANTIGEN ).** | **Non-toxic** | **PROBABLE Non-ALLERGEN** | **Water soluble** |
| **KISSKAGNNYTGEGN** | **0.5772 ( Probable ANTIGEN ).** | **Non-toxic** | **PROBABLE Non-ALLERGEN** | **Water soluble** |
| **KRKDFRWNNARQSDN** | **1.1117 ( Probable ANTIGEN ).** | **Non-toxic** | **PROBABLE Non-ALLERGEN** | **Water soluble** |
| **LNKRKDFRWNNARQS** | **0.9748 ( Probable ANTIGEN ).** | **Non-toxic** | **PROBABLE Non-ALLERGEN** | **Water soluble** |
| **LNPALCDSGKISSKA** | **0.7213 ( Probable ANTIGEN ).** | **Non-toxic** | **PROBABLE Non-ALLERGEN** | **Water soluble** |
| **MDLNPALCDSGKISS** | **0.6950 ( Probable ANTIGEN ).** | **Non-toxic** | **PROBABLE Non-ALLERGEN** | **Water soluble** |
| **NNARQSDNMTNYVGV** | **0.5562 ( Probable ANTIGEN ).** | **Non-toxic** | **PROBABLE Non-ALLERGEN** | **Water soluble** |
| **NNYTGEGNFTPYSND** | **0.9330 ( Probable ANTIGEN ).** | **Non-toxic** | **PROBABLE Non-ALLERGEN** | **Water soluble** |
| **NPALCDSGKISSKAG** | **0.7751 ( Probable ANTIGEN ).** | **Non-toxic** | **PROBABLE Non-ALLERGEN** | **Water soluble** |
| **PALCDSGKISSKAGN** | **0.7578 ( Probable ANTIGEN ).** | **Non-toxic** | **PROBABLE Non-ALLERGEN** | **Water soluble** |
| **PRNYSYMDLNPALCD** | **1.3714 ( Probable ANTIGEN ).** | **Non-toxic** | **PROBABLE Non-ALLERGEN** | **Water soluble** |
| **RKDFRWNNARQSDNM** | **1.0043 ( Probable ANTIGEN ).** | **Non-toxic** | **PROBABLE Non-ALLERGEN** | **Water soluble** |
| **RNYSYMDLNPALCDS** | **1.2025 ( Probable ANTIGEN ).** | **Non-toxic** | **PROBABLE Non-ALLERGEN** | **Water soluble** |
| **RSDCNHVVNTNPRNY** | **0.8276 ( Probable ANTIGEN ).** | **Non-toxic** | **PROBABLE Non-ALLERGEN** | **Water soluble** |
| **SSKAGNNYTGEGNFT** | **0.6914 ( Probable ANTIGEN ).** | **Non-toxic** | **PROBABLE Non-ALLERGEN** | **Water soluble** |
| **SYMDLNPALCDSGKI** | **0.7276 ( Probable ANTIGEN ).** | **Non-toxic** | **PROBABLE Non-ALLERGEN** | **Water soluble** |
| **TGFLNKRKDFRWNNA** | **1.6071 ( Probable ANTIGEN ).** | **Non-toxic** | **PROBABLE Non-ALLERGEN** | **Water soluble** |
| **TITTGFLNKRKDFRW** | **1.6262 ( Probable ANTIGEN ).** | **Non-toxic** | **PROBABLE Non-ALLERGEN** | **Water soluble** |
| **TTGFLNKRKDFRWNN** | **1.7134 ( Probable ANTIGEN ).** | **Non-toxic** | **PROBABLE Non-ALLERGEN** | **Water soluble** |
| **VYDINHGDAQQGVFR** | **0.5614 ( Probable ANTIGEN ).** | **Non-toxic** | **PROBABLE Non-ALLERGEN** | **Water soluble** |
| **YDDTETITTGFLNKR** | **0.7659 ( Probable ANTIGEN ).** | **Non-toxic** | **PROBABLE Non-ALLERGEN** | **Water soluble** |
| **YDINHGDAQQGVFRY** | **0.5855 ( Probable ANTIGEN ).** | **Non-toxic** | **PROBABLE Non-ALLERGEN** | **Water soluble** |
| **YMDLNPALCDSGKIS** | **0.8299 ( Probable ANTIGEN ).** | **Non-toxic** | **PROBABLE Non-ALLERGEN** | **Water soluble** |
| **YSYMDLNPALCDSGK** | **0.8582 ( Probable ANTIGEN ).** | **Non-toxic** | **PROBABLE Non-ALLERGEN** | **Water soluble** |
|  |  |  |  |  |
| **S spike protein** | | | | |
| **Peptide (MHC I)** | **Antigen/Non-Antigen** | **Toxin/Non-Toxin** | **Allergic/non-allergic** | **Solubility** |
| **DKSVPSPLNW** | **0.9531 (Probable ANTIGEN ).** | **Non-Toxin** | **PROBABLE non-ALLERGEN** | **Water soluble** |
| **KSQSSRINF** | **0.4527 (Probable ANTIGEN ).** | **Non-Toxin** | **PROBABLE non-ALLERGEN** | **Water soluble** |
| **LGNKRVELW** | **0.8697 (Probable ANTIGEN ).** | **Non-Toxin** | **PROBABLE non-ALLERGEN** | **Water soluble** |
| **LNDKSVPSPLNW** | **1.3477 (Probable ANTIGEN ).** | **Non-Toxin** | **PROBABLE non-ALLERGEN** | **Water soluble** |
| **NDKSVPSPLNW** | **1.1803 (Probable ANTIGEN ).** | **Non-Toxin** | **PROBABLE non-ALLERGEN** | **Water soluble** |
| **RNMALKGTLLW** | **0.4928 (Probable ANTIGEN ).** | **Non-Toxin** | **PROBABLE non-ALLERGEN** | **Water soluble** |
| **RRFGFTEQF** | **0.5693 (Probable ANTIGEN ).** | **Non-Toxin** | **PROBABLE non-ALLERGEN** | **Water soluble** |
| **SQSSRINF** | **1.1733 (Probable ANTIGEN ).** | **Non-Toxin** | **PROBABLE non-ALLERGEN** | **Water soluble** |
| **SAKSDFMSI** | **1.0041 (Probable ANTIGEN ).** | **Non-Toxin** | **PROBABLE non-ALLERGEN** | **Water soluble** |
| **STTNLDNKLQH** | **0.9549 (Probable ANTIGEN ).** | **Non-Toxin** | **PROBABLE non-ALLERGEN** | **Water soluble** |
| **TNLDNKLQH** | **1.0165 (Probable ANTIGEN ).** | **Non-Toxin** | **PROBABLE non-ALLERGEN** | **Water soluble** |
| **YRNMALKGTL** | **0.5354 (Probable ANTIGEN ).** | **Non-Toxin** | **PROBABLE non-ALLERGEN** | **Water soluble** |
| **TSKSTGPYK** | **0.5424 (Probable ANTIGEN ).** | **Non-Toxin** | **PROBABLE non-ALLERGEN** | **Water soluble** |
| **TTNLDNKLQH** | **0.9169 (Probable ANTIGEN ).** | **Non-Toxin** | **PROBABLE non-ALLERGEN** | **Water soluble** |
|  |  |  |  |  |
| **Peptide (MHC II)** | **Antigen/Non-Antigen** | **Toxin/Non-Toxin** | **Allergic/Non-Allergic** | **Solubility** |
| **SDVGFVEAYNNLEAQ** | **1.0615 (Probable ANTIGEN ).** | **Non-Toxin** | **PROBABLE Non-ALLERGEN** | **Water soluble** |
| **DVGFVEAYNNLEAQA** | **1.0695 ( Probable ANTIGEN )** | **Non-Toxin** | **PROBABLE Non-ALLERGEN** | **Water soluble** |
| **FEPFTVNSVNDSLEP** | **0.8586 (Probable ANTIGEN)** | **Non-Toxin** | **PROBABLE Non-ALLERGEN** | **Water soluble** |
| **TFEPFTVNSVNDSLE** | **0.6872 (Probable ANTIGEN ).** | **Non-Toxin** | **PROBABLE Non-ALLERGEN** | **Water soluble** |
| **PEPITGNKAPDVMLN** | **0.5079 (Probable ANTIGEN ).** | **Non-Toxin** | **PROBABLE Non-ALLERGEN** | **Water soluble** |
| **YYPEPITGNKAPDVM** | **0.6099 (Probable ANTIGEN ).** | **Non-Toxin** | **PROBABLE Non-ALLERGEN** | **Water soluble** |
|  |  |  |  |  |
| **E Envelop protein** | | | | |
| **Peptide (MHC I)** | **Antigen/Non-antigen** | **Toxic/Non-toxic** | **Allergen/Non allergen** | **Solubility** |
| **YNDVKPPVL** | **Probable antigen (0.5212)** | **Non-toxic** | **PROBABLE Non-ALLERGEN** | **Water soluble** |
| **RGRQFYEFY** | **Probable antigen (0.5240)** | **Non-toxic** | **PROBABLE Non-ALLERGEN** | **Water soluble** |
| **YNDVKPPVL** | **Probable antigen (0.5212)** | **Non-toxic** | **PROBABLE Non-ALLERGEN** | **Water soluble** |
| **RQFYEFYNDV** | **Probable antigen (0.5212)** | **Non-toxic** | **PROBABLE Non-ALLERGEN** | **Water soluble** |
| **NDVKPPVL** | **Probable antigen (0.5212)** | **Non-toxic** | **PROBABLE Non-ALLERGEN** | **Water soluble** |
|  |  |  |  |  |
| **Peptide (MHC II)** | **Antigen/Non-antigen** | **Toxic/Non-toxic** | **Allergen/Non allergen** | **Solubility** |
| **QFYEFYNDVKPPVLD** | **Probable non-antigen (0.2130)** | **Non toxin** | **PROBABLE Non-ALLERGEN** | **Water soluble** |
| **RQFYEFYNDVKPPVL** | **Probable non-antigen (-0.0001)** | **Non toxin** | **PROBABLE Non-ALLERGEN** | **Water soluble** |
| **GRQFYEFYNDVKPPV** | **Probable non-antigen (-0.0583)** | **Non toxin** | **PROBABLE Non-ALLERGEN** | **Water soluble** |
| **RGRQFYEFYNDVKPP** | **Probable non-antigen (0.3176)** | **Non toxin** | **PROBABLE Non-ALLERGEN** | **Water soluble** |
|  |  |  |  |  |
| **M membrane protein** | | | | |
| **Peptide (MHC I)** | **Antigen/Non-antigen** | **Toxic/Non-toxic** | **Allergen/Non allergen** | **Solubility** |
| **TQKGSGMDTAL** | **Probable antigen** | **Non-toxin** | **PROBABLE Non-ALLERGEN** | **Water soluble** |
| **TGYSLSDTYK** | **Probable antigen** | **Non-toxin** | **PROBABLE Non-ALLERGEN** | **Water soluble** |
| **RGFLDKIGDTK** | **Probable antigen** | **Non-toxin** | **PROBABLE Non-ALLERGEN** | **Water soluble** |
| **YSLSDTYK** | **Probable antigen** | **Non-toxin** | **PROBABLE Non-ALLERGEN** | **Water soluble** |
| **FLKELGTGY** | **Probable antigen** | **Non-toxin** | **PROBABLE Non-ALLERGEN** | **Water soluble** |
| **KELGTGYSL** | **Probable antigen** | **Non-toxin** | **PROBABLE Non-ALLERGEN** | **Water soluble** |
| **FLKELGTGY** | **Probable antigen** | **Non-toxin** | **PROBABLE Non-ALLERGEN** | **Water soluble** |
| **DTKVGNYRL** | **Probable antigen** | **Non-toxin** | **PROBABLE Non-ALLERGEN** | **Water soluble** |
| **KGSGMDTAL** | **Probable antigen** | **Non-toxin** | **PROBABLE Non-ALLERGEN** | **Water soluble** |
|  |  |  |  |  |
| **Peptide (MHC II)** | **Antigen/Non-antigen** | **Toxic/Non-toxic** | **Allergen/Non allergen** | **Solubility** |
| **LGTGYSLSDTYKRGF** | **Probable antigen** | **Non-toxin** | **PROBABLE Non-ALLERGEN** | **Water soluble** |
|  |  |  |  |  |
| **N Nucleocapsid protein** | | | | |
| **Peptide (MHC I)** | **Antigen/Non-antigen** | **Toxic/Non-toxic** | **Allergen/Non allergen** | **Solubility** |
| **LQRDKVCLL** | **Probable antigen (0.6422)** | **Non-Toxin** | **PROBABLE Non-ALLERGEN** | **Water soluble** |
| **GSLELLSFK** | **Probable antigen (1.0196)** | **Non-Toxin** | **PROBABLE Non-ALLERGEN** | **Water soluble** |
| **SLNLQRDKVCLL** | **Probable antigen (0.7597)** | **Non-Toxin** | **PROBABLE Non-ALLERGEN** | **Water soluble** |
| **RSLNLQRDK** | **Probable antigen (0.4513)** | **Non-Toxin** | **PROBABLE Non-ALLERGEN** | **Water soluble** |
| **RSLNLQRDKVCLL** | **Probable antigen (0.7429)** | **Non-Toxin** | **PROBABLE Non-ALLERGEN** | **Water soluble** |
| **LSFKKERSL** | **Probable antigen (1.4473)** | **Non-Toxin** | **PROBABLE Non-ALLERGEN** | **Water soluble** |
| **EELNPSKLL** | **Probable antigen (0.3676)** | **Non-Toxin** | **PROBABLE Non-ALLERGEN** | **Water soluble** |
| **SLELLSFKKERSL** | **Probable antigen (1.1219)** | **Non-Toxin** | **PROBABLE Non-ALLERGEN** | **Water soluble** |
| **GMILGSLEL** | **Probable antigen (0.8306)** | **Non-Toxin** | **PROBABLE Non-ALLERGEN** | **Water soluble** |
| **LSFKKERSL** | **Probable antigen (1.4473)** | **Non-Toxin** | **PROBABLE Non-ALLERGEN** | **Water soluble** |
| **HPVEPLVQDRV** | **Probable antigen (0.4794)** | **Non-Toxin** | **PROBABLE Non-ALLERGEN** | **Water soluble** |
| **VEELNPSKL** | **Probable antigen (0.6968)** | **Non-Toxin** | **PROBABLE Non-ALLERGEN** | **Water soluble** |
| **ISPTNLEMF** | **Probable antigen (1.5158)** | **Non-Toxin** | **PROBABLE Non-ALLERGEN** | **Water soluble** |
| **SSGSLVTRL** | **Probable antigen (0.5695)** | **Non-Toxin** | **PROBABLE Non-ALLERGEN** | **Water soluble** |
| **FKKERSLNL** | **Probable antigen (1.0177)** | **Non-Toxin** | **PROBABLE Non-ALLERGEN** | **Water soluble** |
| **GVEELNPSKLL** | **Probable antigen (0.5785)** | **Non-Toxin** | **PROBABLE Non-ALLERGEN** | **Water soluble** |
| **LSFKKERSL** | **Probable antigen (1.4473)** | **Non-Toxin** | **PROBABLE Non-ALLERGEN** | **Water soluble** |
|  |  |  |  |  |
| **Peptide (MHC II)** | **Antigen/Non-antigen** | **Toxic/Non-toxic** | **Allergen/Non allergen** | **Solubility** |
| **AHPVEPLVQDRVVEP** | **Probable antigen (0.4284)** | **Non-toxin** | **PROBABLE Non-ALLERGEN** | **Water soluble** |
| **HAHPVEPLVQDRVVE** | **Probable antigen (0.5358)** | **Non-toxin** | **PROBABLE Non-ALLERGEN** | **Water soluble** |
| **LLSFKKERSLNLQRD** | **Probable antigen (0.9174)** | **Non-toxin** | **PROBABLE Non-ALLERGEN** | **Water soluble** |
| **SLNLQRDKVCLLHQE** | **Probable antigen (0.8366)** | **Non-toxin** | **PROBABLE Non-ALLERGEN** | **Water soluble** |
| **RSLNLQRDKVCLLHQ** | **Probable antigen (0.6891)** | **Non-toxin** | **PROBABLE Non-ALLERGEN** | **Water soluble** |
| **LHAHPVEPLVQDRVV** | **Probable antigen (0.5405)** | **Non-toxin** | **PROBABLE Non-ALLERGEN** | **Water soluble** |
| **LLSFKKERSLNLQRD** | **Probable antigen (0.9174)** | **Non-toxin** | **PROBABLE Non-ALLERGEN** | **Water soluble** |
| **HDFTILEQDRMPKTS** | **Probable antigen (0.5653)** | **Non-toxin** | **PROBABLE Non-ALLERGEN** | **Water soluble** |
| **SLNLQRDKVCLLHQE** | **Probable antigen (0.8366)** | **Non-toxin** | **PROBABLE Non-ALLERGEN** | **Water soluble** |
| **LLSFKKERSLNLQRD** | **Probable antigen (0.9174)** | **Non-toxin** | **PROBABLE Non-ALLERGEN** | **Water soluble** |
| **CHDFTILEQDRMPKT** | **Probable antigen (0.4843)** | **Non-toxin** | **PROBABLE Non-ALLERGEN** | **Water soluble** |
| **ELLSFKKERSLNLQR** | **Probable antigen (0.9543)** | **Non-toxin** | **PROBABLE Non-ALLERGEN** | **Water soluble** |
