# Supplementary figures and images for: Machine learning tools used for mapping some immunogenic epitopes within the major structural proteins of the bovine coronavirus (BCoV) and for the in silico design of the multiepitope-based vaccines
Source: Front Vet Sci. 2024 Oct 2;11:1468890. doi: 10.3389/fvets.2024.1468890 (PMC11479863; doi:10.3389/fvets.2024.1468890)

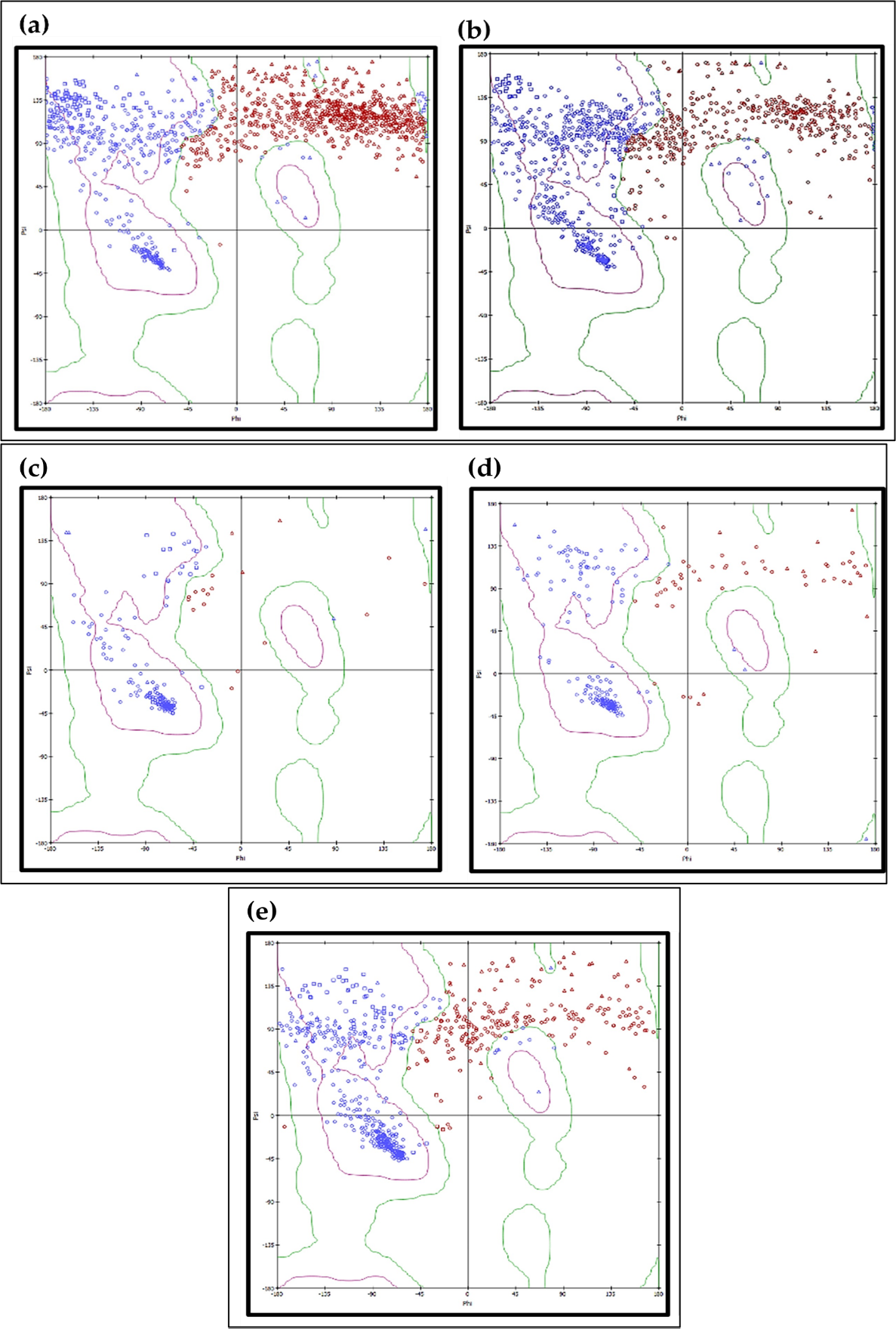

Supplement: Supplementary file 2 [file Data_Sheet_2.ZIP › Supplementary figures in Tiff file/Figure S1.tif]

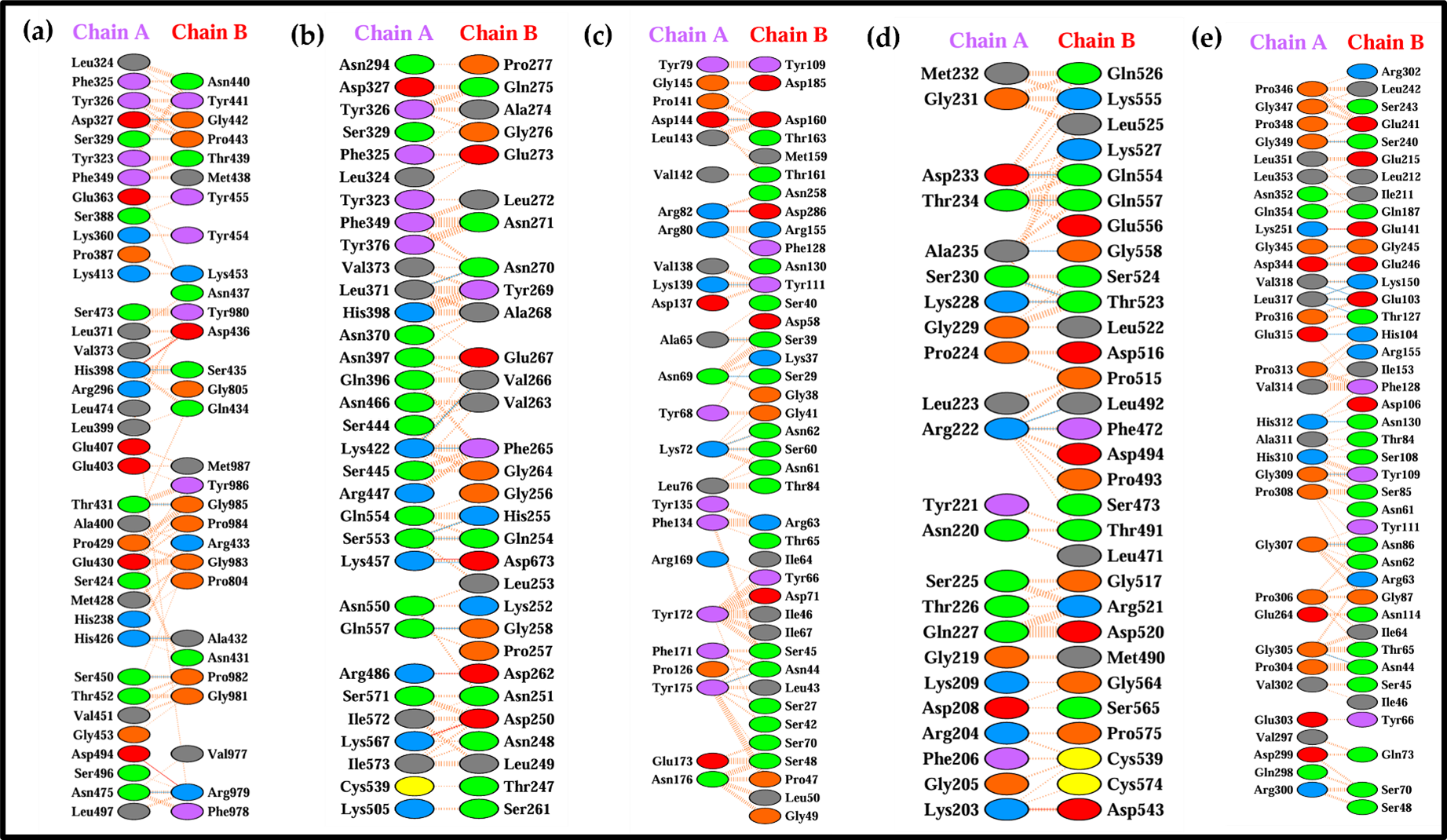

Supplement: Supplementary file 2 [file Data_Sheet_2.ZIP › Supplementary figures in Tiff file/Figure S2 (a).tif]

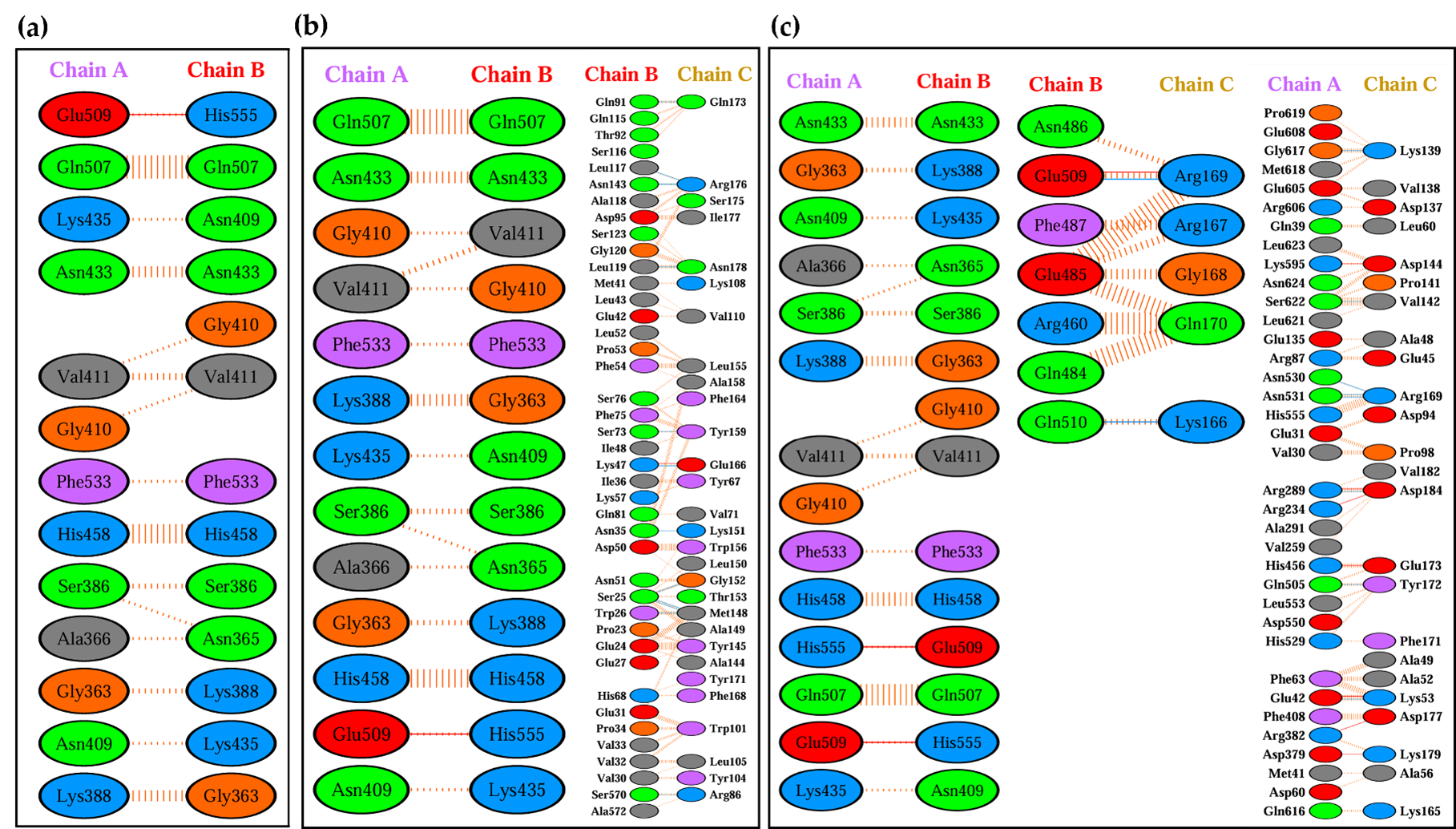

Supplement: Supplementary file 2 [file Data_Sheet_2.ZIP › Supplementary figures in Tiff file/Figure S2 (b).tif]

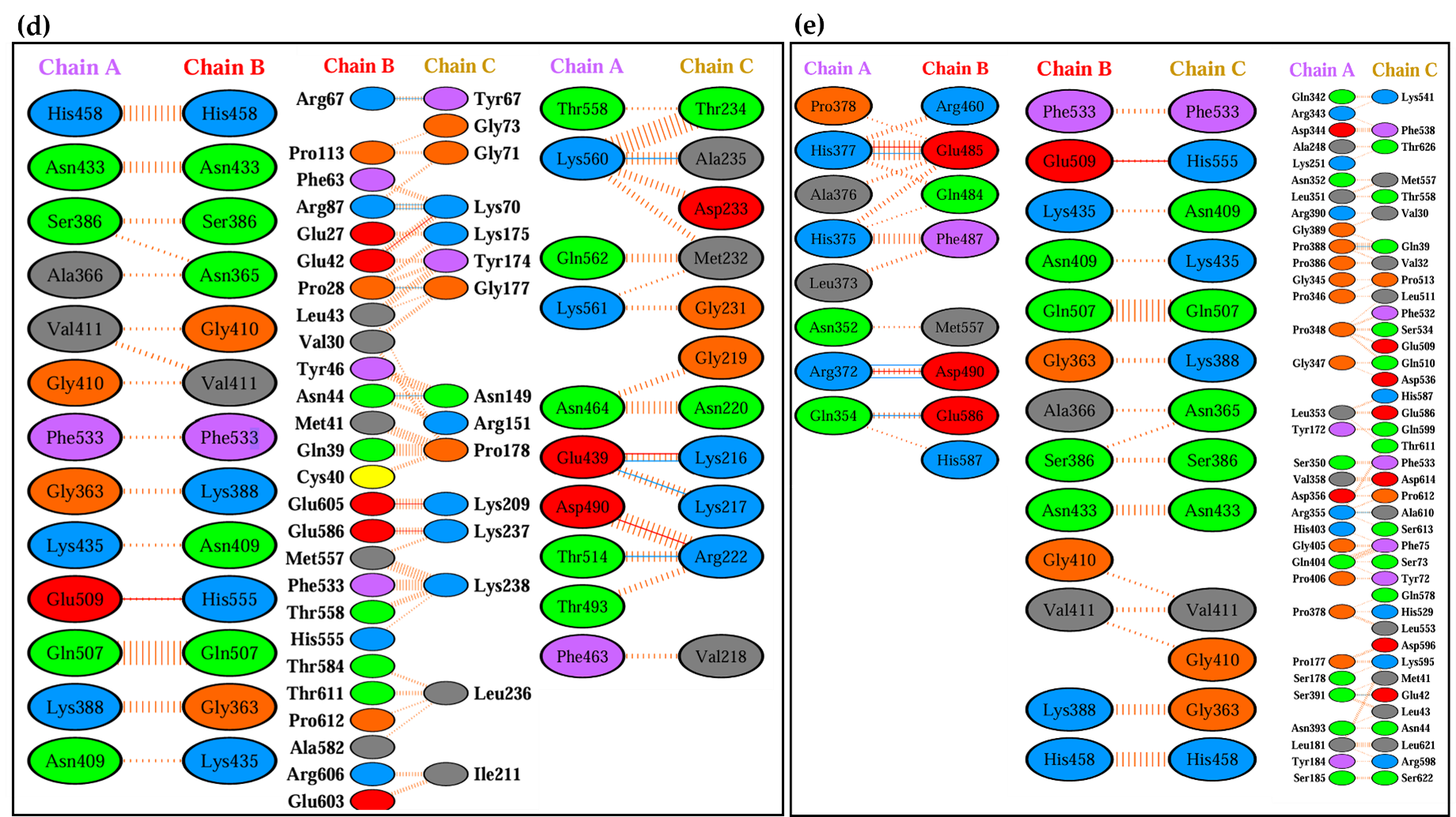

Supplement: Supplementary file 2 [file Data_Sheet_2.ZIP › Supplementary figures in Tiff file/Figure S2 (c).tif]

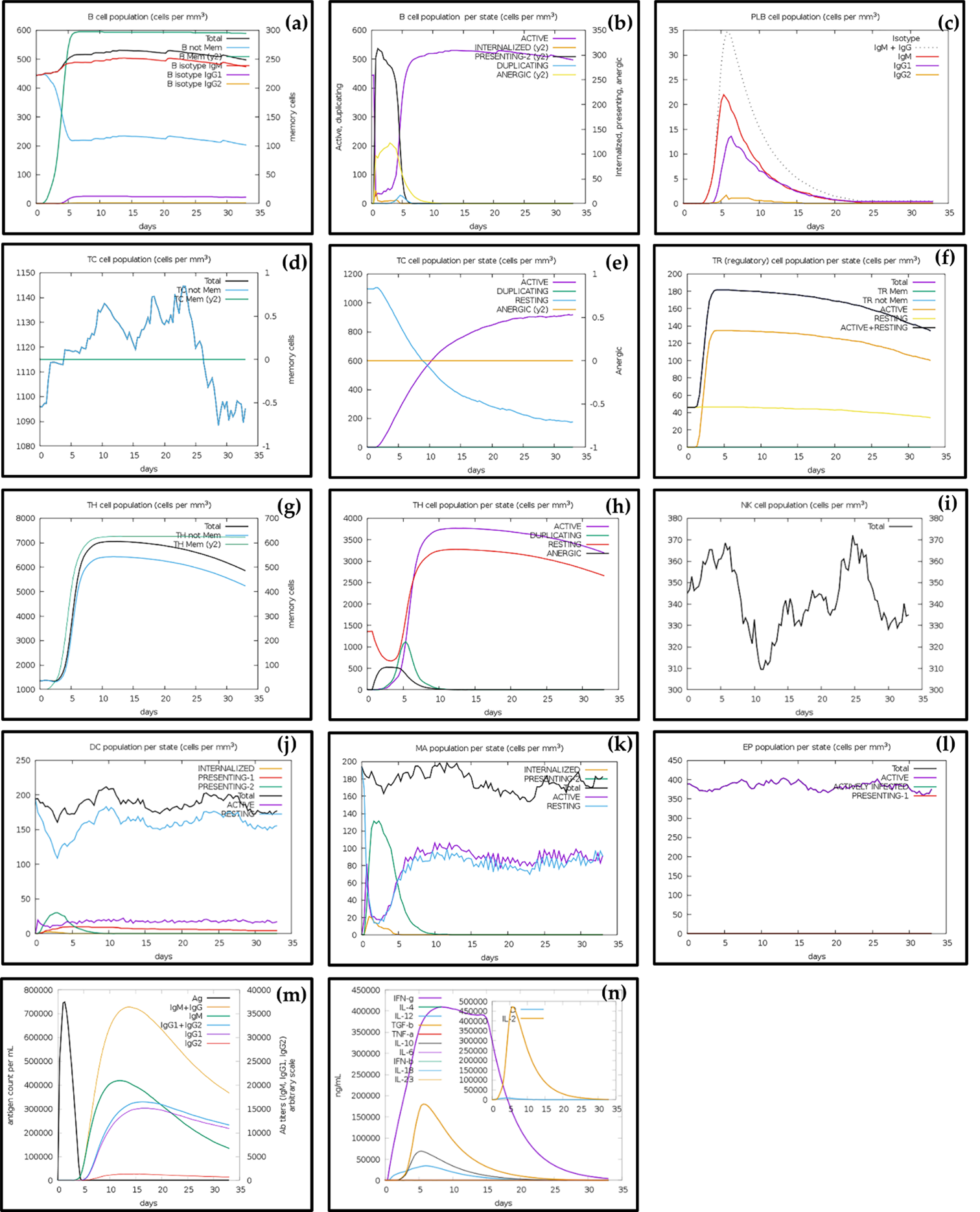

Supplement: Supplementary file 2 [file Data_Sheet_2.ZIP › Supplementary figures in Tiff file/Figure S3 (1).tif]

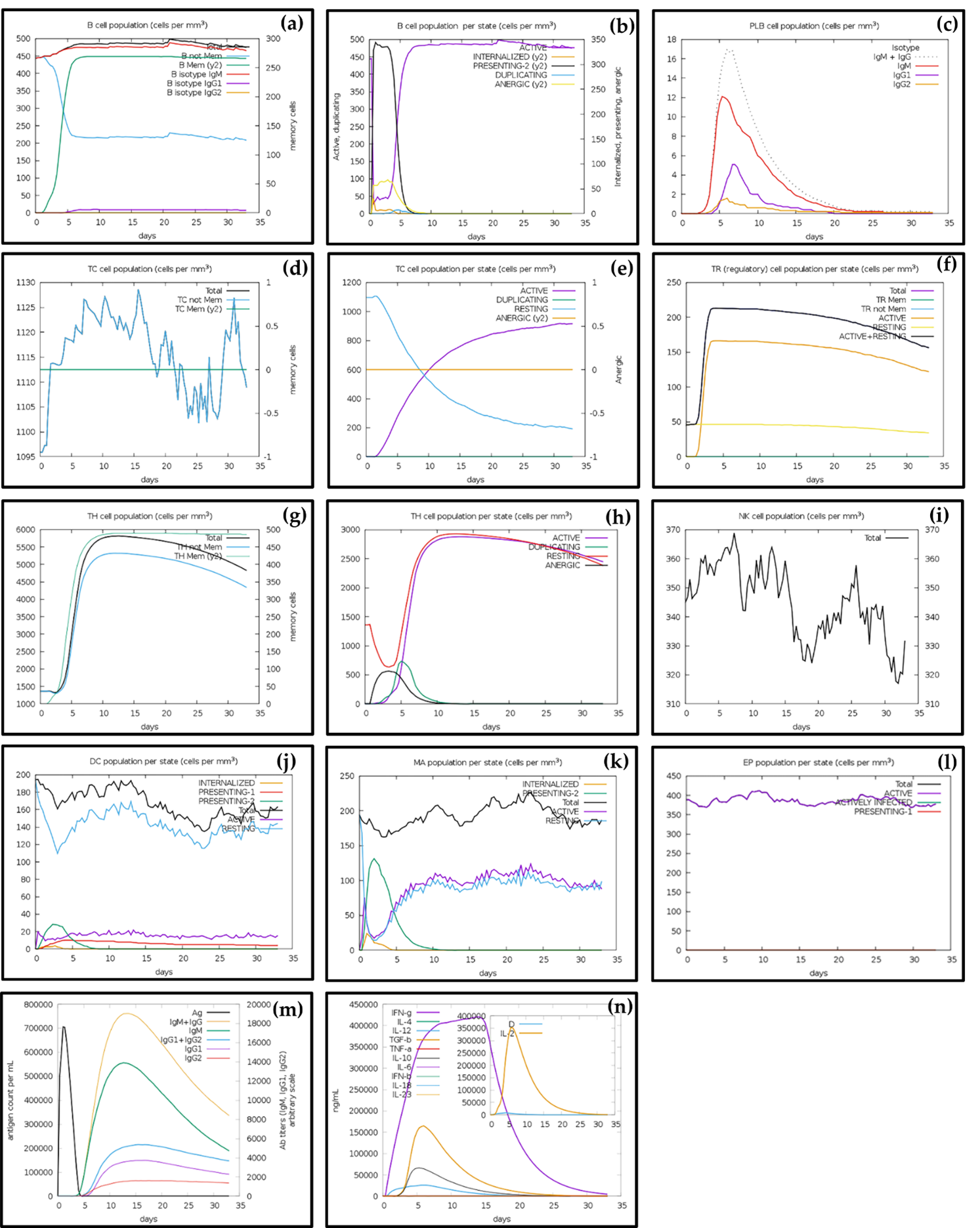

Supplement: Supplementary file 2 [file Data_Sheet_2.ZIP › Supplementary figures in Tiff file/Figure S3 (2).tif]

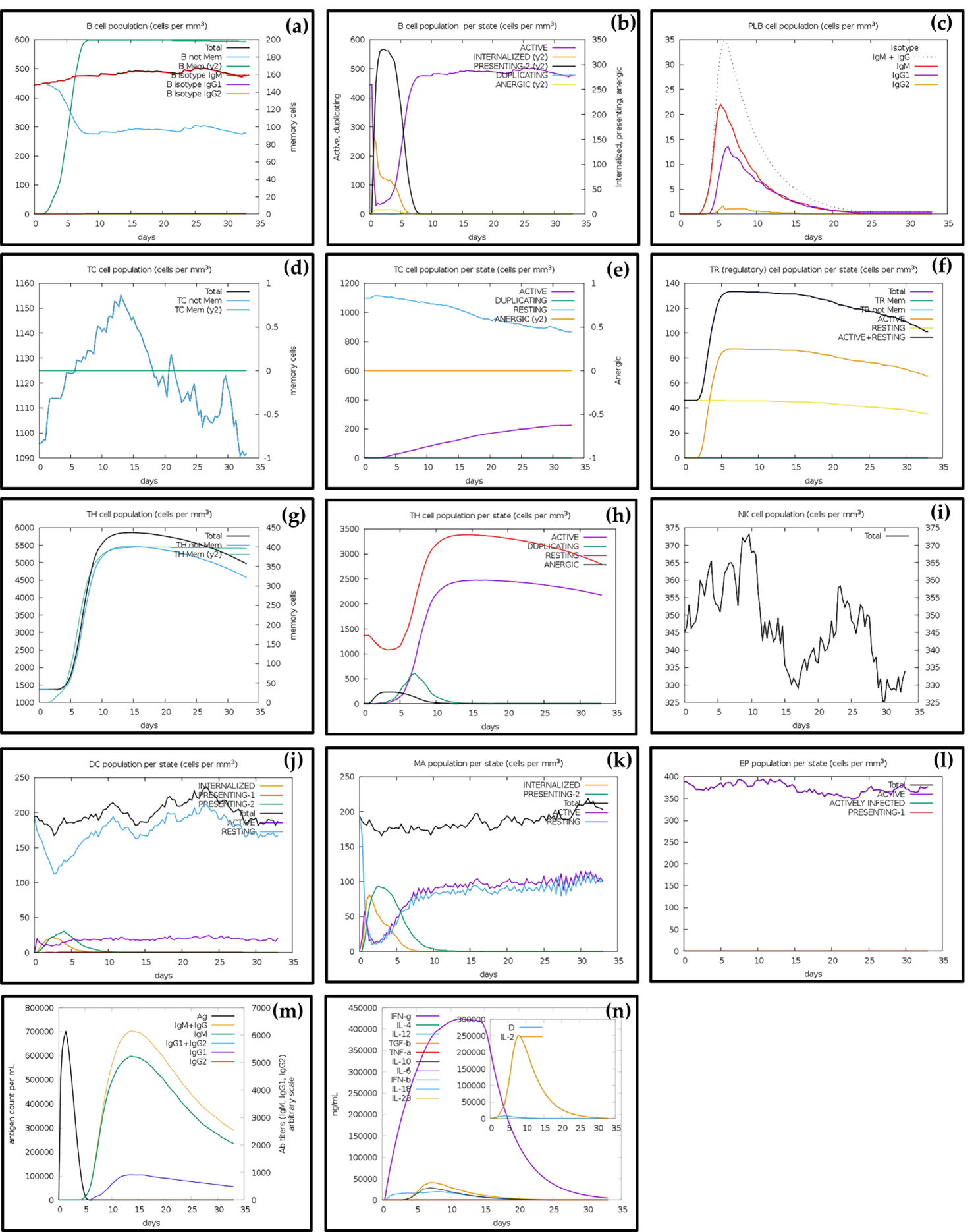

Supplement: Supplementary file 2 [file Data_Sheet_2.ZIP › Supplementary figures in Tiff file/Figure S3 (3).tif]

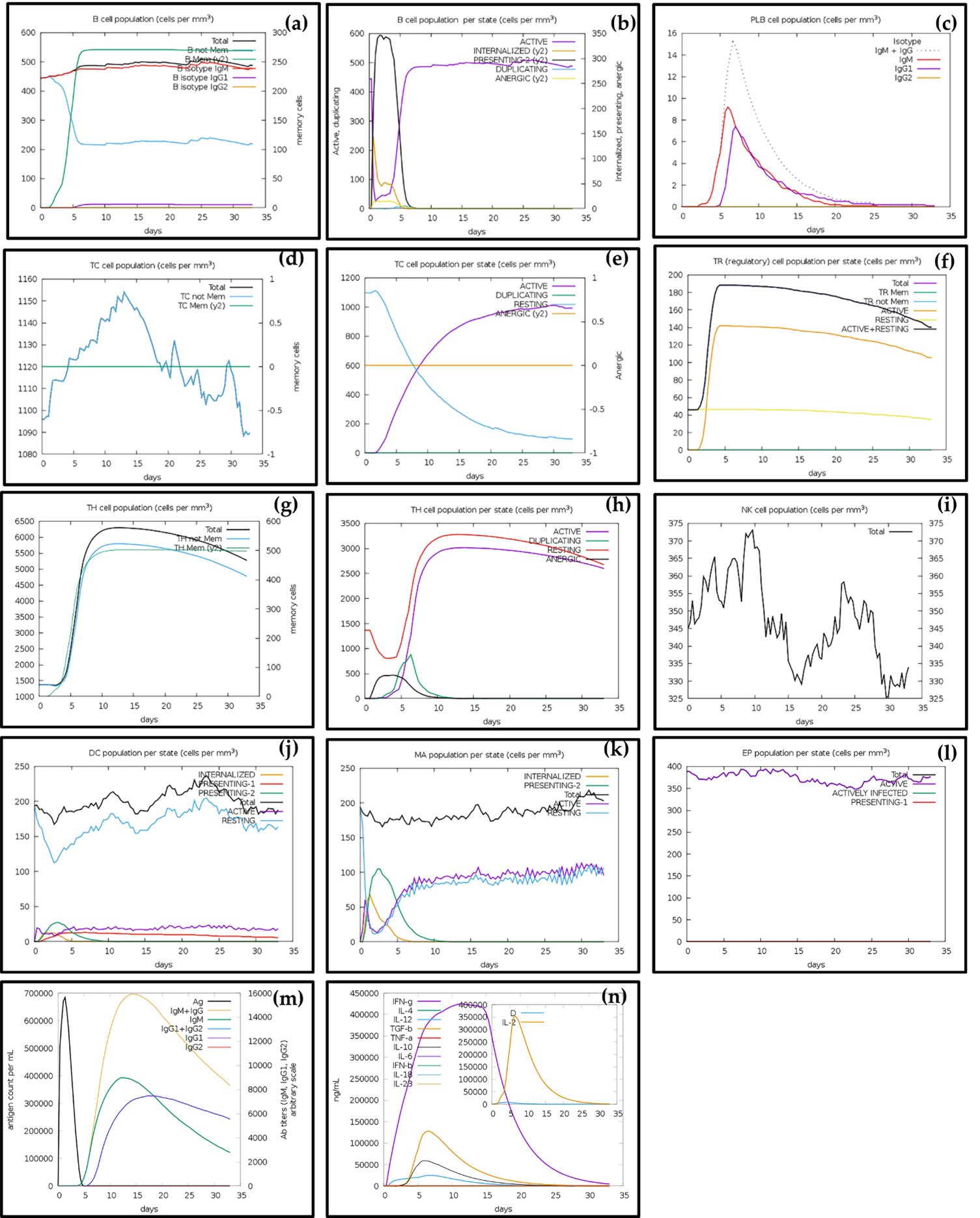

Supplement: Supplementary file 2 [file Data_Sheet_2.ZIP › Supplementary figures in Tiff file/Figure S3 (4).tif]

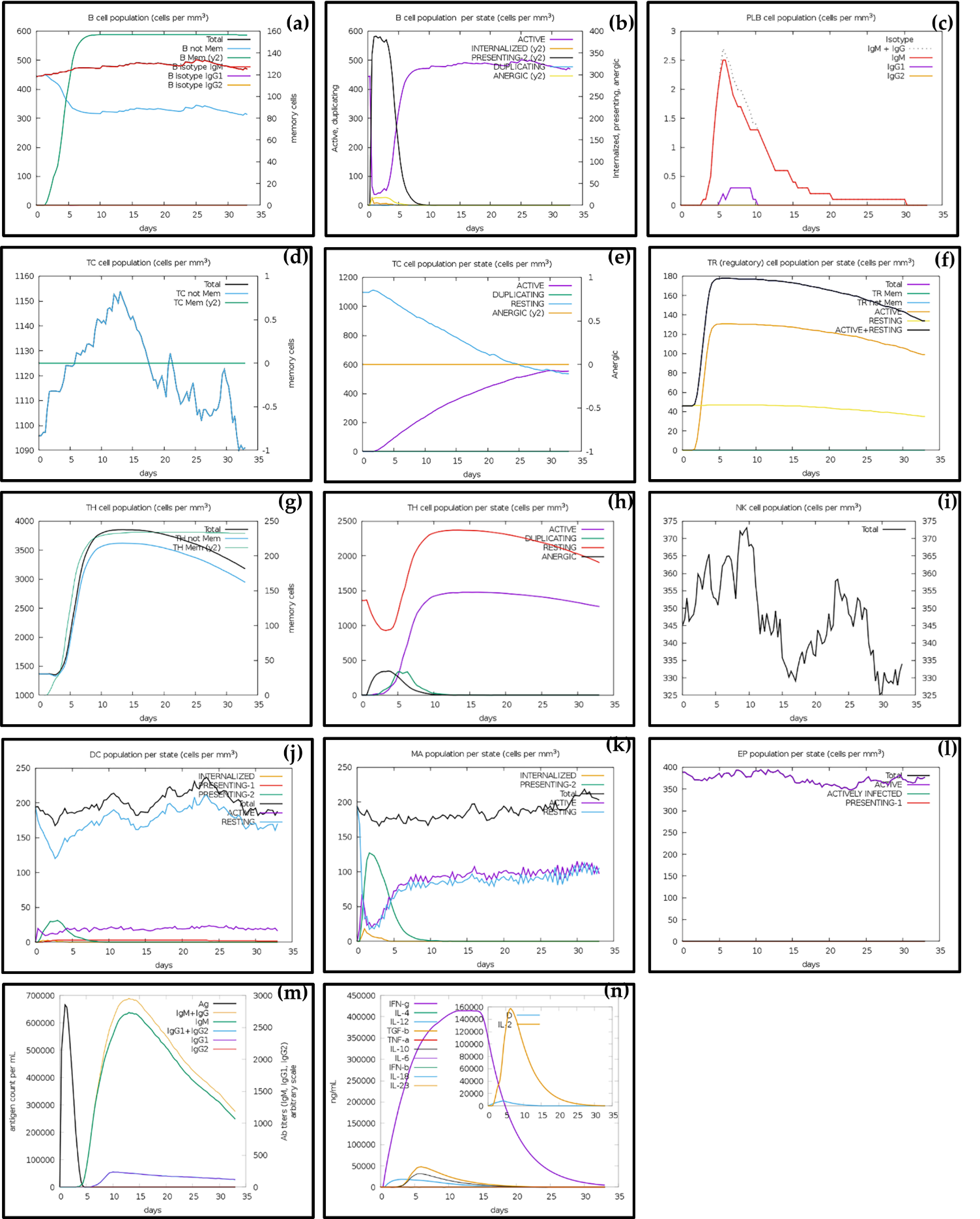

Supplement: Supplementary file 2 [file Data_Sheet_2.ZIP › Supplementary figures in Tiff file/Figure S3 (5).tif]

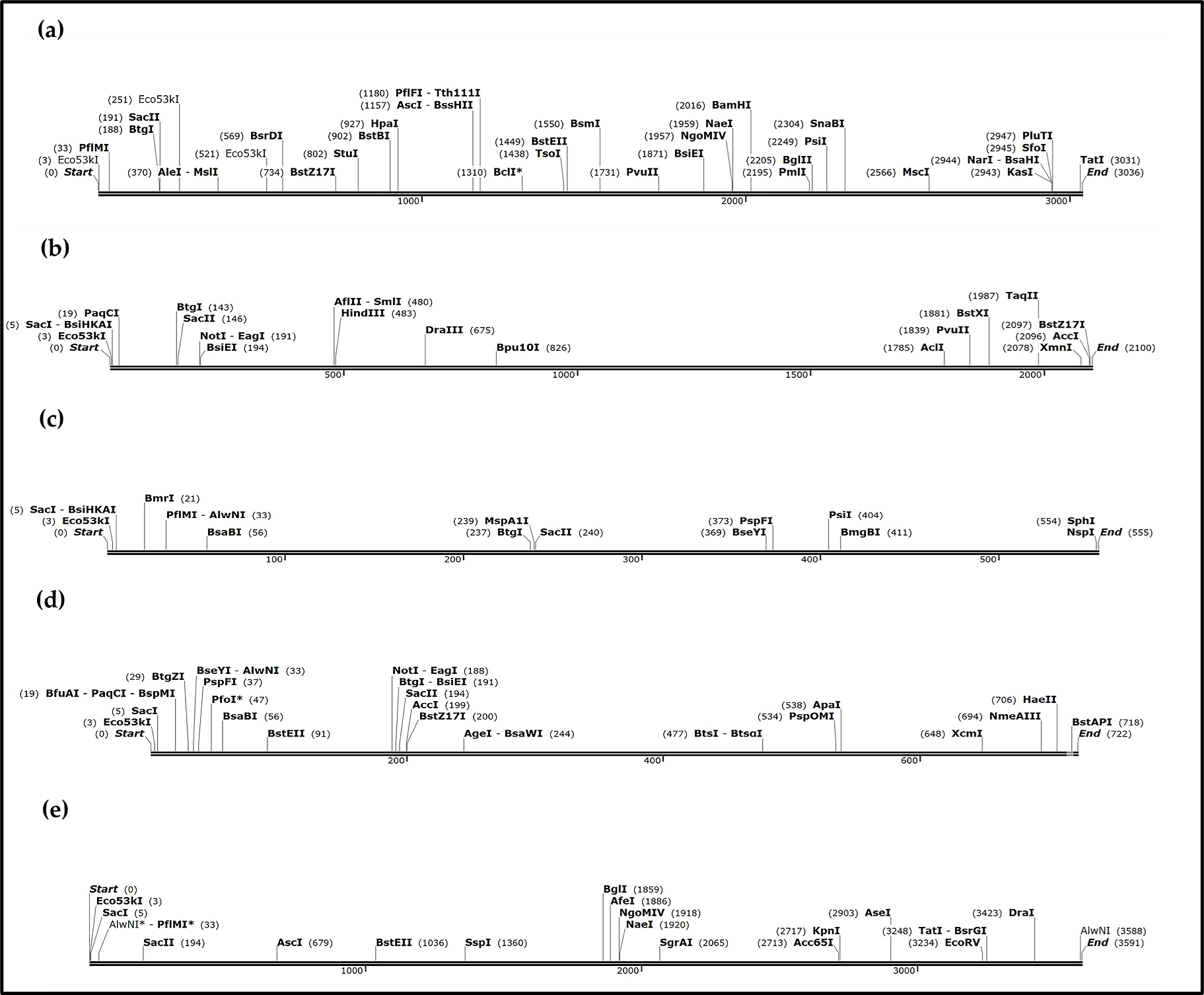

Supplement: Supplementary file 2 [file Data_Sheet_2.ZIP › Supplementary figures in Tiff file/Figure S4 (a-e).tif]

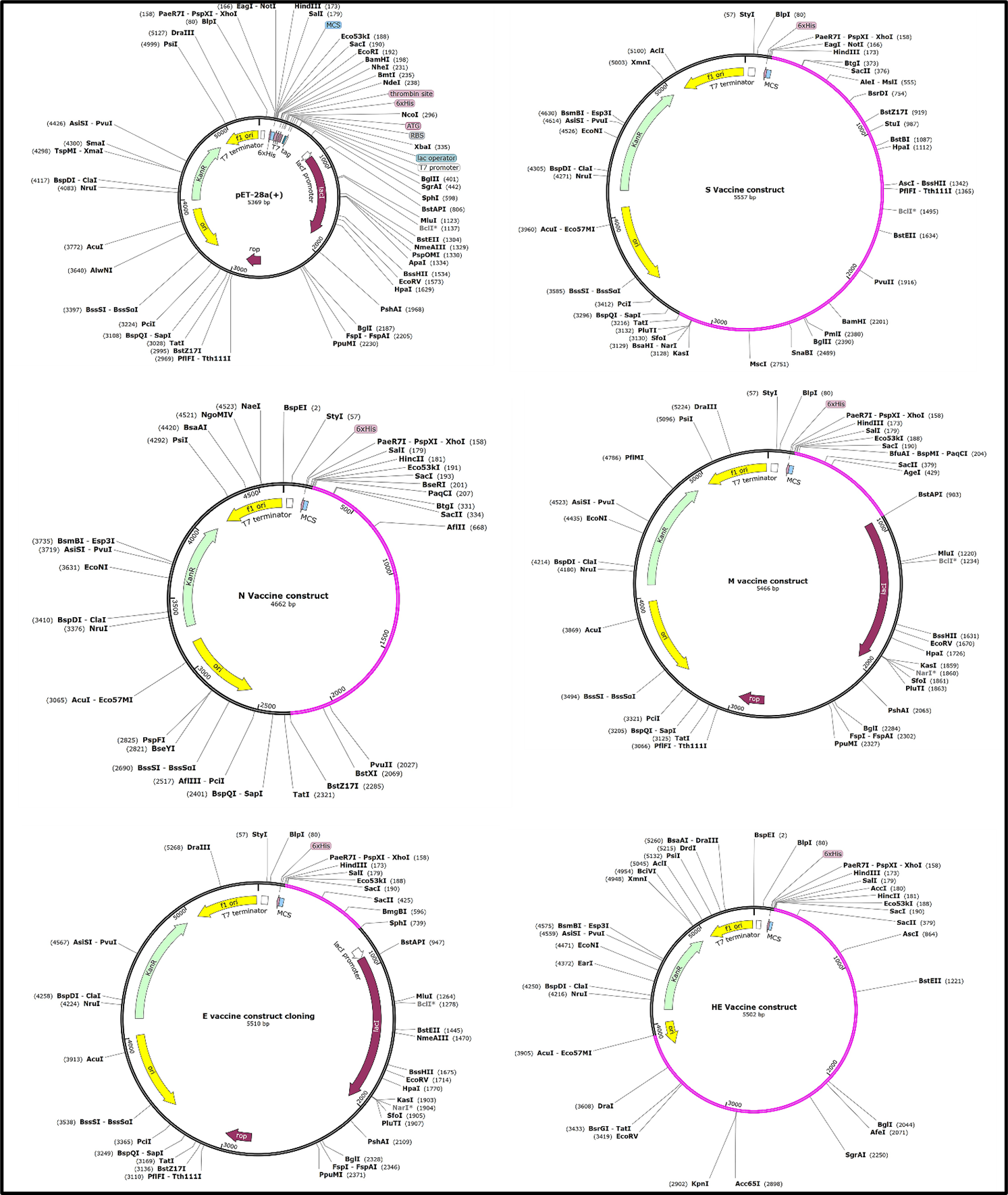

Supplement: Supplementary file 2 [file Data_Sheet_2.ZIP › Supplementary figures in Tiff file/Figure S5 (a-e).tif]
